# A series of Ln_4_^III^ clusters: Dy_4_ single molecule magnet and Tb_4_ multi-responsive luminescent sensor for Fe^3+^, CrO_4_^2−^/Cr_2_O_7_^2−^ and 4-nitroaniline†

**DOI:** 10.1039/c8ra01485j

**Published:** 2018-04-03

**Authors:** Yaru Qin, Yu Ge, Shasha Zhang, Hao Sun, Yu Jing, Yahong Li, Wei Liu

**Affiliations:** College of Chemistry, Chemical Engineering and Materials Science, Soochow University Suzhou 215123 China liyahong@suda.edu.cn

## Abstract

Five tetranuclear lanthanide clusters of compositions [Ln_4_L_4_(NO_3_)_2_(Piv)_2_]·2CH_3_OH (Ln = Gd (1), Tb (2), Dy (3), Ho (4), Er (5); H_2_L = 2-(((2-hydroxy-3-methoxybenzyl)imino)methyl)-6-methoxyphenol; Piv = pivalic acid) were synthesized under solvothermal conditions. The structures of 1–5 were characterized by single-crystal X-ray crystallography. Complexes 1–5 possess a zig-zag topology with [Ln_4_O_6_] cores being formed by the fusion of oxygen atom-bridged two [Ln_2_O_2_] moieties. Direct-current magnetic susceptibility studied in the 2–300 K range revealed weak antiferromagnetic interactions in 1, 2, 4, 5 and ferromagnetic interactions in 3. Complex 3 exhibits single molecule magnet (SMM) behavior. The luminescence studies indicated that complex 2 can serve as highly sensitive and selective luminescent materials for Fe^3+^, CrO_4_^2−^, Cr_2_O_7_^2−^ and 4-nitroaniline (4-NA), demonstrating that complex 2 should be a potential candidate for multi-responsive luminescent sensor.

## Abstract 2

Five tetranuclear lanthanide clusters were synthesized. Dy_4_ complex exhibits single molecule magnet (SMM) behavior and Tb_4_ compound shows sensing properties towards Fe^3+^, CrO_4_^2−^, Cr_2_O_7_^2−^ and 4-nitroaniline (4-NA).

## Graphical abstract

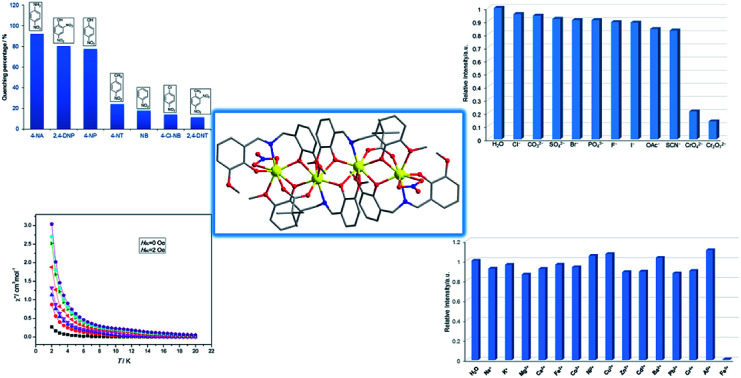

## Introduction

Polynuclear lanthanide clusters have been attracting considerable attention due to their fascinating structures^1^ as well as potential applications in single molecule magnets (SMMs),^2^ luminescent devices^3^ and magnetocaloric materials.^4^ SMMs are of considerable promise as molecular spintronic devices for high-density data storage.^5^ The significant anisotropy of lanthanides arising from large unquenched orbital angular momentum^2*a*,2*u*^ has made lanthanides to be attractive candidates for SMMs. Recently, the research efforts towards lanthanide SMMs have been devoted to polynuclear complexes with variable nuclearities.^2^ Moreover, owing to the characteristic sharp emission peaks, a large Stokes shift and a wide emission range, the utilization of lanthanide complexes as luminescent materials, particularly luminescent sensor,^6^ also attracts intensive interest.

Fe^3+^ ion plays an important role in industry and in various metabolic processes.^7^ However, excessive Fe^3+^ ion probably leads to some diseases, such as Alzheimer's disease, due to production of reactive oxygen species (ROS).^8^ CrO_4_^2−^ and Cr_2_O_7_^2−^ ions can cause severe environment pollution,^9^ gemmulation, renal failure, lung cancer, and skin allergy by prolonged exposure.^10^ Nitroaromatic compounds (NACs), *e.g.*, nitroaromatic (NB), 4-nitrophenol (4-NP), 4-nitroaniline (4-NA), 4-nitrotoluene (4-NT), 4-nitrochlorobenzene (4-Cl-NB), 2,4-dinitrotoluene (2,4-DNT), 2,4-dinitrophenol (2,4-DNP), *etc.*, are threatening human life.^11^ Thus, it is of high importance to develop fast and facile methods for detecting those ions and compounds. A number of fluorescence-based sensing materials based on metal–organic frameworks (MOFs),^12^ coordination polymers,^13^ and mononuclear compounds^14^ have been utilized to detect specific target inorganic anions, cations or organic molecules, due to high efficiency, selectivity and simplicity. Whereas, luminescent sensors base on lanthanide polynuclear clusters were rarely studied. The recent advances in this area indicated that they are potential sensors for nitroaromatic explosive compounds^15^ and F^−^ ion.^16^

We are interested in designing and synthesizing Ln^III^ clusters supported by *o*-vanillin-containing Schiff base ligand 2-(((2-hydroxy-3-methoxybenzyl)imino)methyl)-6-methoxyphenol (H_2_L) and pivalic acid. The selection of both H_2_L and pivalic acid as the ligands is based on the following considerations. (i) The combination of the *o*-vanillin-based ligand and pivalic acid fosters the formation of polynuclear clusters owing to the incorporation of the phenolic oxygen atoms from the H_2_L ligand with the carboxylate group from pivalic acid. (ii) H_2_L is an electron rich π-conjugated multidentate organic ligand, which can act as antennae to sensitize the weak luminescent metal centres, such as Tb(iii). (iii) Although the single molecule magnet behaviours of a series of Ln^III^ clusters have been studied, their multiple fluorescence detection abilities towards Fe^3+^, CrO_4_^2−^/Cr_2_O_7_^2−^, and NACs have not been reported. In this work, five tetranuclear lanthanide clusters of compositions [Ln_4_L_4_(NO_3_)_2_(Piv)_2_]·2CH_3_OH (Ln = Gd (1), Tb (2), Dy (3), Ho (4), Er (5)) supported by H_2_L were synthesized *via* solvothermal method. Complexes 1–5 possess [Ln_4_O_6_] cores formed by the fusion of two phenoxide oxygen-bridged two [Ln_2_O_2_] moieties. The magnetic studies indicated antiferromagnetic interactions in 1, 2, 4 and 5. Complex 3 exhibits typical single molecule magnet behaviour. The luminescent sensing properties of 2 for selective and sensitive detections of Fe^3+^, CrO_4_^2−^/Cr_2_O_7_^2−^ ions in aqueous solutions and 4-NA in ethanol solutions were also investigated. The low detection limits and high quenching constants *K*_SV_ indicate that complex 2 may potentially be considered as a multi-responsive luminescence-based sensor for quantitative and highly sensitive detections of 4-NA, Fe^3+^ and CrO_4_^2−^/Cr_2_O_7_^2−^ ions.

## Experimental section

### Synthesis of 2-(((2-hydroxy-3-methoxybenzyl)imino)methyl)-6-methoxyphenol (H_2_L)

**Scheme 1:**
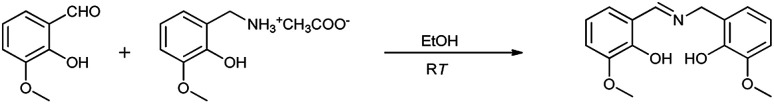
Synthesis of the H_2_L ligand.

All solvents and regents were of analytical grade and used as received without further purification. 3-Methoxysalicylamine was prepared according to the reported procedure.^17^ A solution of methoxysalicylaldehyde (20 mmol) in ethanol was added to a solution of 3-methoxysalicylamine (20 mmol) in 15 mL ethanol. After the reaction mixture was stirred at room temperature for 4 hours, yellow precipitates were formed. The yellow precipitates were isolated, washed with hexane and dried in vacuum to afford the product as a yellow solid (Scheme 1). Yield: 4.76 g (83%). ^1^H NMR (600 MHz, chloroform-d) *δ* 8.38 (s, 1H), 6.90–6.84 (m, 3H), 6.83–6.74 (m, 3H), 4.82 (s, 2H), 3.87 (s, 3H), 3.85 (s, 3H). ^13^C NMR (151 MHz, chloroform-d) *δ* 165.57, 152.66, 148.63, 146.39, 143.48, 123.64, 123.01, 121.34, 119.66, 118.56, 117.50, 113.90, 109.89, 56.60, 56.06, 56.04. Elemental anal. calcd for C_16_H_17_NO_4_: C, 66.89; H, 5.96; N, 4.88%. Found: C, 66.77; H, 6.23; N, 5.18%. Selected IR data (KBr, cm^−1^): 3400(w), 1630(m), 1593(w), 1487(m), 1474(s), 1461(m), 1440(m), 1351(w), 1315(w), 1277(m), 1249(s), 1237(s), 1216(m), 1188(m), 1162(m), 1074(m), 993(m), 979(m), 903(m), 842(w), 830(w), 801(w), 785(w), 738(s), 760(s), 743(s), 734(s), 710(w).

### Physical measurement

Elemental analyses for C, H, and N were carried out with a Perkin-Elmer 2400 analyser. Fourier transform (FT) IR spectra were recorded with a Bruker VERTEX 70 FTIR spectrophotometer in the range of 600–4000 cm^−1^. Magnetic susceptibility measurements were performed in the temperature range of 2–300 K, using a Quantum Design MPMS XL-7 SQUID magnetometer. Powder X-ray diffraction (PXRD) was obtained from a Rigaku D/Max-2500 diffractometer at 40 kV and 100 mA with a Cu-target tube and a graphite monochromator. Fluorescence spectra for the samples were recorded on a FLS-920 fluorescence spectrophotometer. The UV-Vis absorption spectra were determined with an Agilent Cary-60 spectra photometer at room temperature. X-ray photoelectron spectroscopy (XPS) experiments were carried out by a ESCALAB 250Xi spectrometer using an Al Kα source at room temperature.

### X-ray single-crystal data collection and structure determination

The diffraction data for 1–5 were collected on a Bruker D8-VENTURE (120 K) CCD X-ray diffractometer equipped with a graphite monochromated Mo Kα radiation (*λ* = 0.71073 Å). The *ω*–2*θ* scan technique was applied. The crystal structures of all complexes were solved with the Olex2 solve solution program^18^ using Intrinsic Phasing and refined by full-matrix least-squares minimization using the ShelXL refinement package.^19^ All H atoms were placed on appropriate positions in theory and their positions were refined using the riding model. The details of the crystal parameters, data collection and refinements for the complexes are summarized in Table 1. Selected bond lengths and angles with their estimated standard deviations are listed in Table S1.†

**Table 1:** Crystal data and structure refinements for 1–5

	1	2	3	4	5
Empirical formula	C_76_H_86_N_6_O_28_Gd_4_	C_76_H_86_N_6_O_28_Tb_4_	C_76_H_86_N_6_O_28_Dy_4_	C_76_H_86_N_6_O_28_Ho_4_	C_76_H_86_N_6_O_28_Er_4_
Formula weight	2160.50	2167.18	2181.50	2191.02	2200.54
Temperature/K	120	120	120	120	120
Crystal system	Monoclinic	Monoclinic	Monoclinic	Monoclinic	Monoclinic
Space group	*P*2_1_/*c*	*P*2_1_/*c*	*P*2_1_/*c*	*P*2_1_/*c*	*P*2_1_/*c*
*a*/Å	13.4900(07)	13.4681(15)	13.457(03)	13.4321(16)	13.4131(7)
*b*/Å	11.9674(06)	11.9193(13)	11.905(02)	11.8480(14)	11.8637(7)
*c*/Å	24.5060(13)	24.483(3)	24.500(06)	24.450(3)	24.4580(14)
*α*/°	90	90	90	90	90
*β*/°	95.961(2)	95.984(4)	96.03	96.123(4)	96.222(2)
*γ*/°	90	90	90	90	90
Volume/Å^3^	3934.9(9)	3908.9(7)	3903.1(07)	3868.9(8)	3869.1(4)
*Z*	2	2	2	2	2
*ρ*_calc_ g cm^−3^	1.823	1.841	1.856	1.881	1.889
*μ*/mm^−1^	3.413	3.661	3.872	4.133	4.381
*F*(000)	2128.0	2136.0	2144.0	2152.0	2160.0
Crystal size/mm^3^	02 × 0.04 × 0.04	0.4 × 0.05 × 0.05	0.40 × 0.05 × 0.05	0.40 × 0.05 × 0.05	0.25 × 0.24 × 0.23
*θ* Range	2.28 to 27.502	2.287 to 27.565	2.928 to 27.488	2.501 to 27.63	2.394 to 27.605
Index ranges	−17 ≤ *h* ≤ 17, −15 ≤ *k* ≤ 15, −31 ≤ *l* ≤ 29	−17 ≤ *h* ≤ 17, −15 ≤ *k* ≤ 15, −31 ≤ *l* ≤ 31	−17 ≤ *h* ≤ 17, −15 ≤ *k* ≤ 15, −26 ≤ *l* ≤ 31	−17 ≤ *h* ≤ 17, −15 ≤ *k* ≤ 15, −31 ≤ *l* ≤ 31	−17 ≤ *h* ≤ 16, −15 ≤ *k* ≤ 15, −28 ≤ *l* ≤ 31
Reflections collected	59 585	60 747	53 678	59 835	60 923
Independent reflections	9047 [*R* (int) = 0.0954]	8987 [*R* (int) = 0.0456]	8994 [*R* (int) = 0.0408]	8938 [*R* (int) = 0.0803]	8920 [*R* (int) = 0.0483]
Data/restraints/parameters	9047/18/533	8987/12/533	8944/12/533	8938/12/533	8920/12/533
Goodness-of-fit on *F*^2^	1.093	1.116	1.182	1.058	1.206
Final *R* indexes [*I* ≥ 2σ (I)]	*R*_1_ = 0.0428, w*R*_2_ = 0.0616	*R*_1_ = 0.0267, w*R*_2_ = 0.0513	*R*_1_ = 0.0311, w*R*_2_ = 0.0531	*R*_1_ = 0.0313, w*R*_2_ = 0.0603	*R*_1_ = 0.0329, w*R*_2_ = 0.0546
Final *R* indexes [all data]	*R*_1_ = 0.0737, w*R*_2_ = 0.0682	*R*_1_ = 0.0405, w*R*_2_ = 0.0575	*R*_1_ = 0.0383, w*R*_2_ = 0.0549	*R*_1_ = 0.0500, w*R*_2_ = 0.0685	*R*_1_ = 0.0413, w*R*_2_ = 0.0563
Largest diff. peak/hole/e Å^−3^	0.81/−0.88	0.90/−0.77	0.85/−1.08	0.85/−1.10	0.93/−1.41

### Synthesis of 1–5

A mixture of Ln(NO_3_)_3_·6H_2_O (Ln = Gd, Tb, Dy, Ho, Er) (0.1 mmol), H_2_L (0.1 mmol), pivalic acid (0.1 mmol), triethylamine (0.2 mmol) and CH_3_OH (2.5 mL) was sealed in a pyrex-tube (10 mL). The tube was heated for 24 h at 80 °C. After cooling to room temperature, yellow block crystals suitably for X-ray diffraction analysis were obtained.

#### [Gd_4_L_4_(NO_3_)_2_(Piv)_2_]·2CH_3_OH (1)

Yield of 1 is 47% (0.0254 g) based on Gd. Elemental anal. calcd for C_76_H_86_N_6_O_28_Gd_4_: C, 42.25; H, 4.01; N, 3.89%. Found: C, 42.44; H, 3.99; N, 4.12%. Selected IR data (KBr, cm^−1^): 2955(m), 1621(m), 1548(m), 1468(s), 1420(m), 1403(m), 1323(w), 1296(w), 1271(m), 1245(s), 1228(m), 1166(m), 1062(m), 1028(m), 1013(m), 977(w), 951(w), 914(w), 867(m), 785(m), 735(s), 612(w).

#### [Tb_4_L_4_(NO_3_)_2_(Piv)_2_]·2CH_3_OH (2)

Yield of 2 is 53% (0.0287 g) based on Tb. Elemental anal. calcd for C_76_H_86_N_6_O_28_Tb_4_: C, 42.12; H, 4.00; N, 3.88%. Found: C, 41.74; H, 4.04; N, 4.07%. Selected IR data (KBr, cm^−1^): 2956(m), 1622(m), 1549(m), 1469(s), 1420(m), 1402(m), 1323(w), 1288(w), 1271(m), 1245(s), 1229(m), 1166(m), 1063(m), 1028(m), 1013(m), 978(w), 952(w), 914(w), 868(m), 785(m), 735(s), 612(w).

#### [Dy_4_L_4_(NO_3_)_2_(Piv)_2_]·2CH_3_OH (3)

Yield of 3 is 54% (0.0294 g) based on Dy. Elemental anal. calcd for C_76_H_86_N_6_O_28_Dy_4_: C, 41.84; H, 3.97; N, 3.85%. Found: C, 41.84; H, 4.05; N, 3.95%. Selected IR data (KBr, cm^−1^): 2956(m), 1623(m), 1550(m), 1470(s), 1420(m), 1403(m), 1324(w), 1289(w), 1271(m), 1245(s), 1230(m), 1166(m), 1063(m), 1029(m), 1014(m), 979(w), 953(w), 916(w), 868(m), 785(m), 735(s), 613(w).

#### [Ho_4_L_4_(NO_3_)_2_(Piv)_2_]·2CH_3_OH (4)

Yield of 4 is 53% (0.0290 g) based on Ho. Elemental anal. calcd for C_76_H_86_N_6_O_28_Ho_4_: C, 41.66; H, 3.96; N, 3.84%. Found: C, 41.47; H, 4.05; N, 4.00%. Selected IR data (KBr, cm^−1^): 2956(m), 1623(m), 1551(m), 1471(s), 1421(m), 1403(m), 1325(w), 1290(w), 1272(m), 1245(s), 1231(m), 1166(m), 1063(m), 1030(m), 1014(m), 979(w), 953(w), 916(w), 868(m), 785(m), 736(s), 613(w).

#### [Er_4_L_4_(NO_3_)_2_(Piv)_2_]·2CH_3_OH (5)

Yield of 5 is 51% (0.0280 g) based on Er. Elemental anal. calcd for C_76_H_86_N_6_O_28_Er_4_: C, 41.48; H, 3.94; N,3.82%. Found: C, 40.83; H, 4.03; N 3.93%. Selected IR data (KBr, cm^−1^): 2956(m), 1623(m), 1552(m), 1471(s), 1421(m), 1402(m), 1326(w), 1290(w), 1272(m), 1243(s), 1231(m), 1166(m), 1063(m), 1030(m), 1014(m), 980(w), 953(w), 916(w), 868(m), 785(m), 736(s), 613(w).

## Results and discussion

### Synthesis of complexes

The tetranuclear complexes 1–5 were synthesized under solvothermal conditions. When a mixture of Ln(NO_3_)_3_·6H_2_O (Ln = Gd, Tb, Dy, Ho, Er), H_2_L (the synthesis of H_2_L was shown in Scheme 1), and pivalic acid, in a 1 : 1 : 1 molar ratio in MeOH/Et_3_N was sealed in a Pyrex-tube and heated under solvothermal conditions, yellow block crystals were generated after being heated at 353 K for one day. The experimental powder X-ray diffraction patterns matched well with those simulated from the crystal structures, demonstrating the purity of complexes 1–5.

### Description of crystal structure of 1–5

**Fig. 1:**
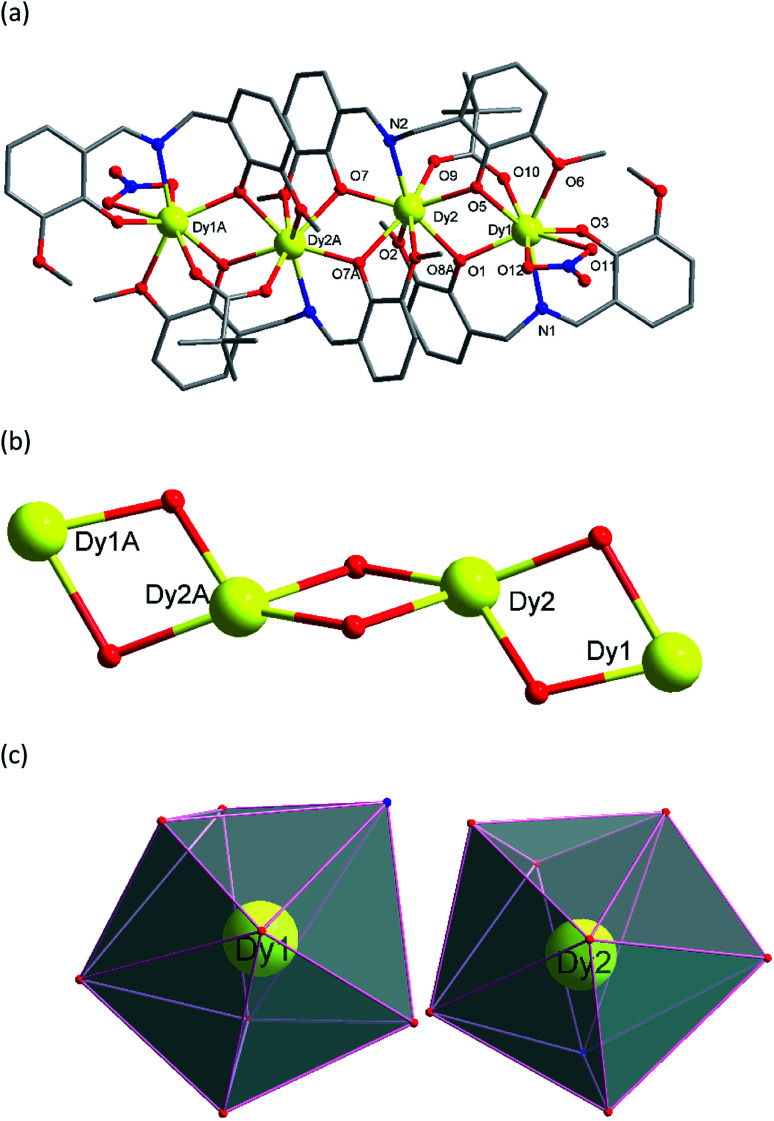
(a) Molecular structure of 3. Symmetry code: *A* = 2 − X, −Y, 1 − Z (hydrogen atoms and solvents have been omitted for clarity). (b) The tetranuclear [Dy_4_O_6_] core in 3. (c) Coordination polyhedrons of Dy(iii) ions in 3.

**Scheme 2:**
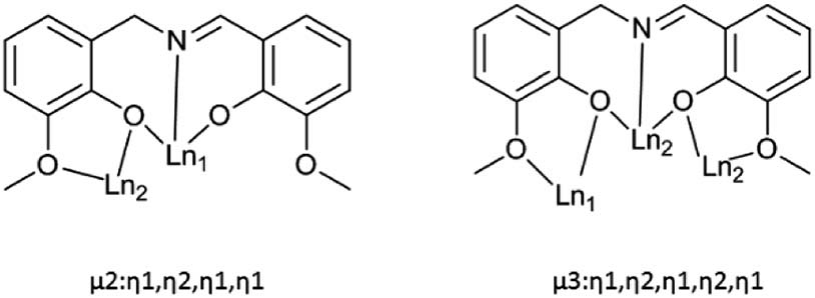
The coordination modes of ligand (L^2−^) in complexes 1–5.

Single crystal X-ray analyses and powder X-ray diffraction analyses indicated that complexes 1–5 are isostructural. They crystalize in the monoclinic *P*2_1_/*c* space group (Table 1, Fig. 1). Thus, complex 3 was chosen as a representative for 1–5 to describe the structure in detail. The molecular structure of 3 contains four Dy(iii) ions in a zig-zag topology, four L^2−^ ligands, two deprotonated pivalic acids, two NO_3_^−^ anions and two CH_3_OH solvate molecules (Fig. 1). Two L^2−^ ligands coordinate in a *μ*_2_: *η*^1^, *η*^2^, *η*^1^, *η*^1^ fashion whereas the other two L^2−^ ligands coordinate in a *μ*_3_: *η*^1^, *η*^2^, *η*^1^, *η*^2^, *η*^1^ mode (Scheme 2). The structure of 3 is composed by two asymmetric [Dy2] units which are connected by phenolate bridges from the Schiff base ligands with Dy⋯Dy distances of 3.697(2) Å (Dy1⋯Dy2) and 3.839(2) Å (Dy2⋯Dy2A). Dy1 centre is coordinated by two oxygen atoms (O1 and O3) from one ligand, one nitrogen atom (N1) from the same ligand, two oxygen atoms (O5 and O6) from another ligand set, two oxygen atoms (O11 and O12) from one NO_3_^−^ ion, and one oxygen atom (O10) from a deprotonated pivalic acid, forming a NO7 coordination environment. Dy2 centre was coordinated by two oxygen atoms (O7 and O9) from one ligand, one nitrogen atom (N2) from the same ligand, four oxygen atoms (O7A, O8A, O1 and O2) from another two ligand sets and one oxygen atom (O9) from deprotonated pivalic acid. It also forms a NO7 coordination environment. The Dy1–O bond lengths are in the range of 2.169(3)–2.534(3) Å and the Dy1–N bond is 2.491(3) Å. The Dy2–O bond lengths are in the range of 2.249(3)–2.635(3) Å and the Dy2–N bond is 2.443(3) Å. They are all comparable to those of reported complexes with [DyNO7] units.^2*o*,20^ The exact coordination geometries of the octa-coordinated Ln(iii) ions were analysed by SHAPE 2.1 software^21^ and resulting data were shown in Table S2.† The resulting data from the closer analysis reveal that Ln1(iii) ion is in a square antiprism (*D*_4d_) configuration while Ln2(iii) is in a biaugmented trigonal prism (*C*_2V_) configuration with a minimum continuous shape measures (CShM) value.

### Magnetic studies

**Fig. 2:**
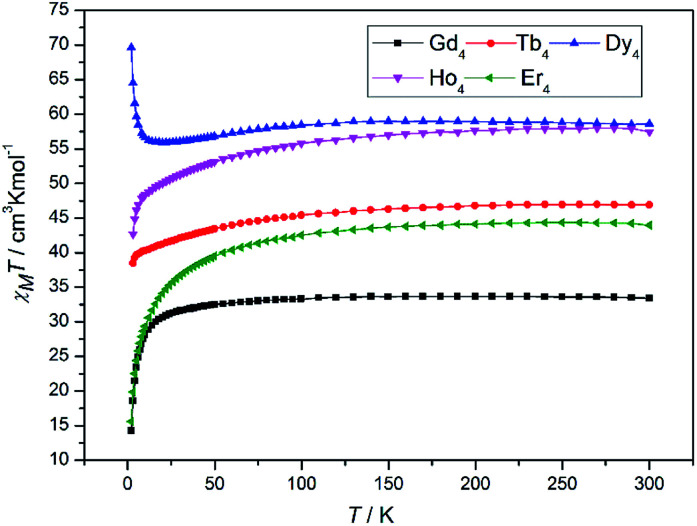
Temperature dependence of the *χ*_M_*T* products in the range of 2–300 K under 1000 Oe for 1–5.

The magnetic properties of 1–5 were investigated by direct-current (DC) magnetic susceptibility studies under a magnetic field of 1000 Oe in the temperature range 2–300 K. The experimental *χ*_M_*T* values at 300 K and magnetic behaviours of 1–5 are shown in Table 2. The *χ*_M_*T versus T* plots for 1–5 are shown in Fig. 2. For 1, the room temperature *χ*_M_*T* value of 33.41 cm^3^ K mol^−1^ is slightly higher than value of 31.52 cm^3^ K mol^−1^ expected for four magnetically isolated Gd^III^ ions (^8^S_7/2_, *g* = 2). At 300 K, the *χ*_M_*T* values of 46.91 and 58.50 cm^3^ K mol^−1^ for complexes 2 and 3 are close to the expected values of 47.20 cm^3^ K mol^−1^ for uncoupled Tb^III^ ions (^7^F_6_, *g* = 2) and 56.68 cm^3^ K mol^−1^ for uncoupled Dy^III^ ions (^8^H_15/2_, *g* = 4/3). The room temperature *χ*_M_*T* values for 4 and 5 are 57.47 and 44.21 cm^3^ K mol^−1^, respectively, which are slightly lower than values of 58.28 cm^3^ K mol^−1^ expected for four magnetically uncoupled Ho^III^ ions (^5^I_8_, *g* = 5/4) and 45.92 cm^3^ K mol^−1^ expected for four magnetically uncoupled Er^III^ ions (^4^I_15/2_, *g* = 6/5). Upon cooling, the *χ*_M_*T* product of 1 stays almost constant in the temperature range of 300–30 K and then decreases rapidly, reaching minimum value of 13.80 cm^3^ K mol^−1^ at 2 K. This magnetic behaviour suggests weak antiferromagnetic exchange interactions. The application of a Curie–Weiss law gives values of *θ* = −1.98 K, *C* = 32.86 for 1 (Fig. S6†). With the decrease of temperature, the *χ*_M_*T* values for 2, 4 and 5 firstly decrease slowly and then decline sharply to minimum values at 2 K. The decrease of *χ*_M_*T* values for 2, 4 and 5 is typical for Ln^III^ ions and is due to several factors, namely, the thermal depopulation of the excited *m*_J_ sublevels of the ^2*s*+1^*Γ*_J_ ground state of the Ln^III^ ion originated by a crystal field symmetry, in combination with the weak Ln^III^–Ln^III^ antiferromagnetic interactions in 2, 4 and 5.^22^

**Table 2:** Direct-current (dc) magnetic susceptibilities of 1–5

Complex	Metal ions	Theoretical *χ*_M_*T*/cm^3^ K mol^−1^	Experimental *χ*_M_*T*/cm^3^ K mol^−1^ at 300 K	Magnetic behaviour
1	Gd	31.52	33.41	AF
2	Tb	47.20	46.91	AF
3	Dy	58.68	58.50	F
4	Ho	58.28	57.47	AF
5	Er	45.92	44.21	AF

Upon cooling, the *χ*_M_*T* value of 3 decreases imperceptibly and reaches minimum value of 55.9 cm^3^ K mol^−1^ at 23 K, which is attributed by the thermal depopulation of Stark effect for a single Dy^III^ ion. Continuous being cooled down to 2 K, the *χ*_M_*T* product sharply increases to a maximum value of 69.6 cm^3^ K mol^−1^. This magnetic phenomenon may be caused by the presence of ferromagnetic interactions between the spin carriers.^2*g*,23^

**Fig. 3:**
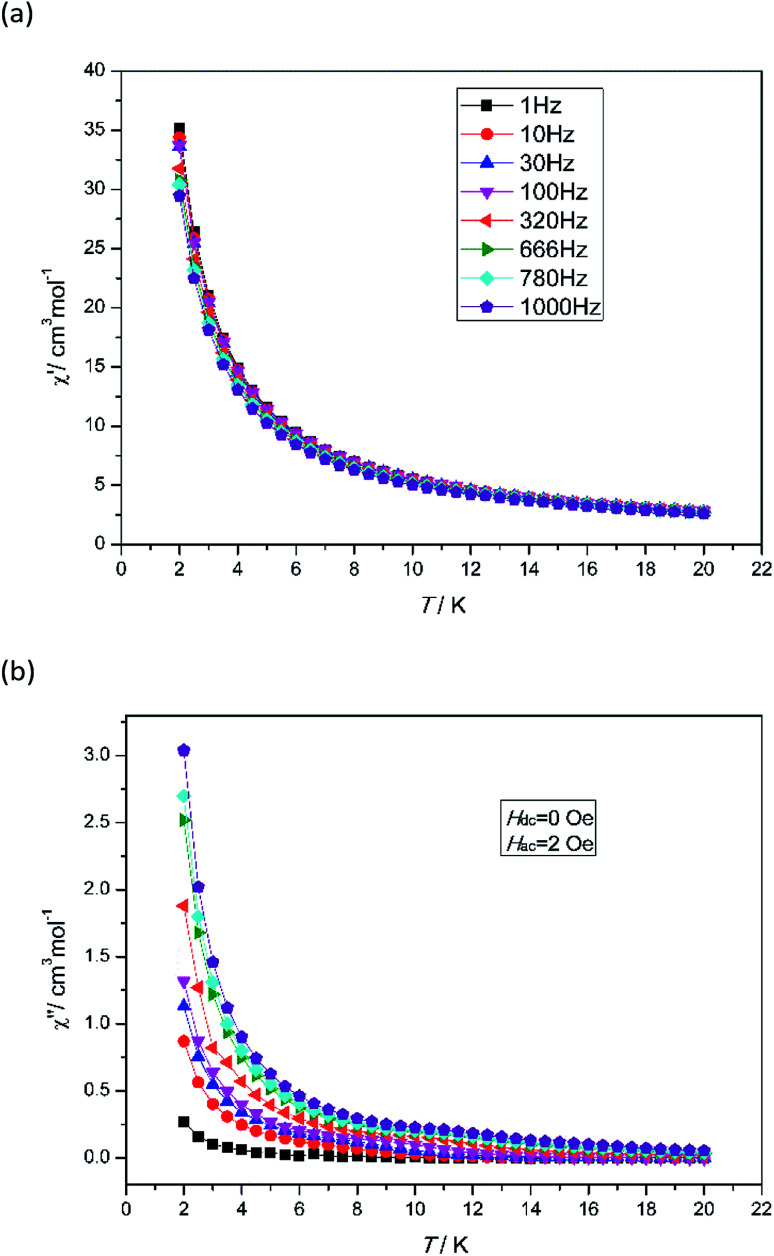
Temperature dependence of the in-phase *χ*′ (a) and out-of-phase *χ*′′ (b) for 3 under zero dc field at different frequencies in the range of 2–20 K.

**Fig. 4:**
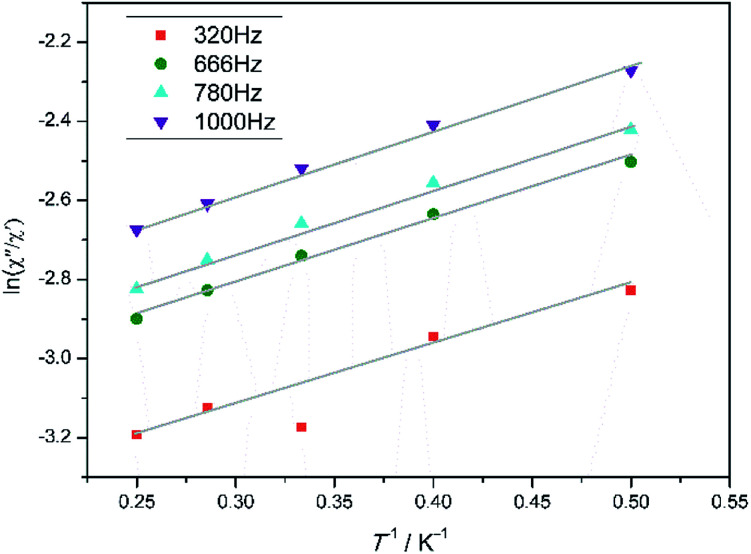
Plots of ln (*χ*′′/*χ*′) *vs. T*^−1^ for 3 in the range 2–4.0 K. The grey solid lines correspond to the best fit result.

To investigate the dynamic magnetic properties of 3, alternating-current (ac) magnetic susceptibilities were determined under zero-dc field with a 2 Oe oscillating ac field (Fig. 3). The out-of-phase susceptibility (*χ*′′) displays frequency-dependent phenomenon at low temperatures, which suggests the presence of slow relaxation of the magnetization, typical of SMM behaviour.^24^ However, the peak maxima are not found. Ac susceptibility measurements of 3 under 0–10 000 Oe dc field at 1000 Hz were conducted (Fig. S7†). Unfortunately, no optimum field was found. The energy barrier (*Ea*/*K*_B_) and pre-exponential factor (*τ*_0_) values were calculated from the frequency-dependent ac susceptibility data by using the Debye model based on the equation ln(*χ*′′/*χ*′) = ln(*ωτ*_0_) + *Ea*/*K*_B_*T*.^24*a*^ The best fitting results give *Ea*/*K*_B_ ≈ 1.61 K and *τ*_0_ ≈ 7.42 × 10^−6^ s (Fig. 4). Apparently, the *τ*_0_ value is comparable to the expected values 10^−6^ to 10^−11^ for typical SMMs.^25^

### Luminescent properties

The solid-state luminescent properties of the H_2_L ligand and 2 were measured at room temperature (Fig. S8†). The H_2_L ligand displays a broad emission around 544 nm (*λ*_ex_ = 467 nm), which is due to the π → π* transition between the ligand orbitals. Complex 1 exhibits a ligand-centred broad band emission at 557 nm and a shoulder at 483 nm (*λ*_ex_ = 396 nm), which is attributed to the ligand centred π–π* and charge–transfer transition between ligands and metal centers^26^ (Fig. S8b†). As shown in Fig. S8,† the solid-state emission spectra of 3–5 (Dy_4_, Ho_4_, Er_4_) display emission bands centred at 482, 482, 480 nm respectively under excitation at 324 nm. The blue shifts of 3–5 are tentatively assigned to the electrostatic interaction between the ligand and metal ions.^26*b*^ Complex 2 displays four characteristic emission peaks at 490, 544, 584, and 618 nm (*λ*_ex_ = 360 nm), corresponding to ^5^D_4_ → ^7^F_6_, ^5^D_4_ → ^7^F_5_, ^5^D_4_ → ^7^F_4_ and ^5^D_4_ → ^7^F_3_,^3*b*^ respectively (Fig. S9†). The strongest emission of 2 at 544 nm is responsible for the green emission, which can be observed with the naked eyes. The π-conjugated multidentate organic ligand can act as antennae to sensitize the weak luminescent of Tb(iii). Therefore, the strong and sharp luminescence emission of 2 can be visually observed, which allows it to be considered as a fluorescent sensor. The results of the luminescence sensing experiments revealed that complex 2 can be served as a luminescent senior for selectively sensing 4-NA, Fe^3+^, CrO_4_^2−^ and Cr_2_O_7_^2−^.

In order to examine the stability, the PXRD patterns of 2 were measured after being soaked in aqueous solutions with different pH values for several days. The PXRD patterns remained almost unaltered and agreed well with the simulated pattern, revealing the excellent stability of 2 in different solutions (Fig. S10 and S11†). Twenty samples of 2 (2 mg/2 ml suspension) after detection of 4-NA (2 mL, 1 × 10^−3^), Fe^3+^ (1 mL, 3 × 10^−3^), CrO_4_^2−^ (2 mL, 2 × 10^−3^) and Cr_2_O_7_^2−^ (2 mL, 2 × 10^−3^) were filtered and dried to recover for PXRD test.

### Detection of metal ions

Based on the fact that compound 2 possesses excellent stability in the aqueous solutions with broad pH range, its potential florescent sensing properties towards metal ions were investigated. Firstly, a series of aqueous solutions of 2 were prepared by adding 2 mg of complex 2 into 2 mL of deionized water. Then, the resulting suspensions were sonicated for 8, 10 and 30 minutes, respectively. The sizes of suspensions of particles of 2 were analysed by scanning electron microscopy. The size-distribution histograms were obtained from measuring the diameters of 100 randomly selected particles. According to the histograms of the particle size distributions in Fig. S12,† the average diameters of 2 after being sonicated for 8, 15 and 30 minutes are estimated to be approximately 15.58 ± 3.02, 12.75 ± 2.30 and 7.77 ± 2.62 μm, respectively, of which the sample (2) after being sonicated for 30 minutes displays the smallest particle size. The smaller size of the 2 leading to larger surface areas and more accessible active sites on their surface would be beneficial to realize highly sensitive and fast-response luminescent sensing.^27^

**Fig. 5:**
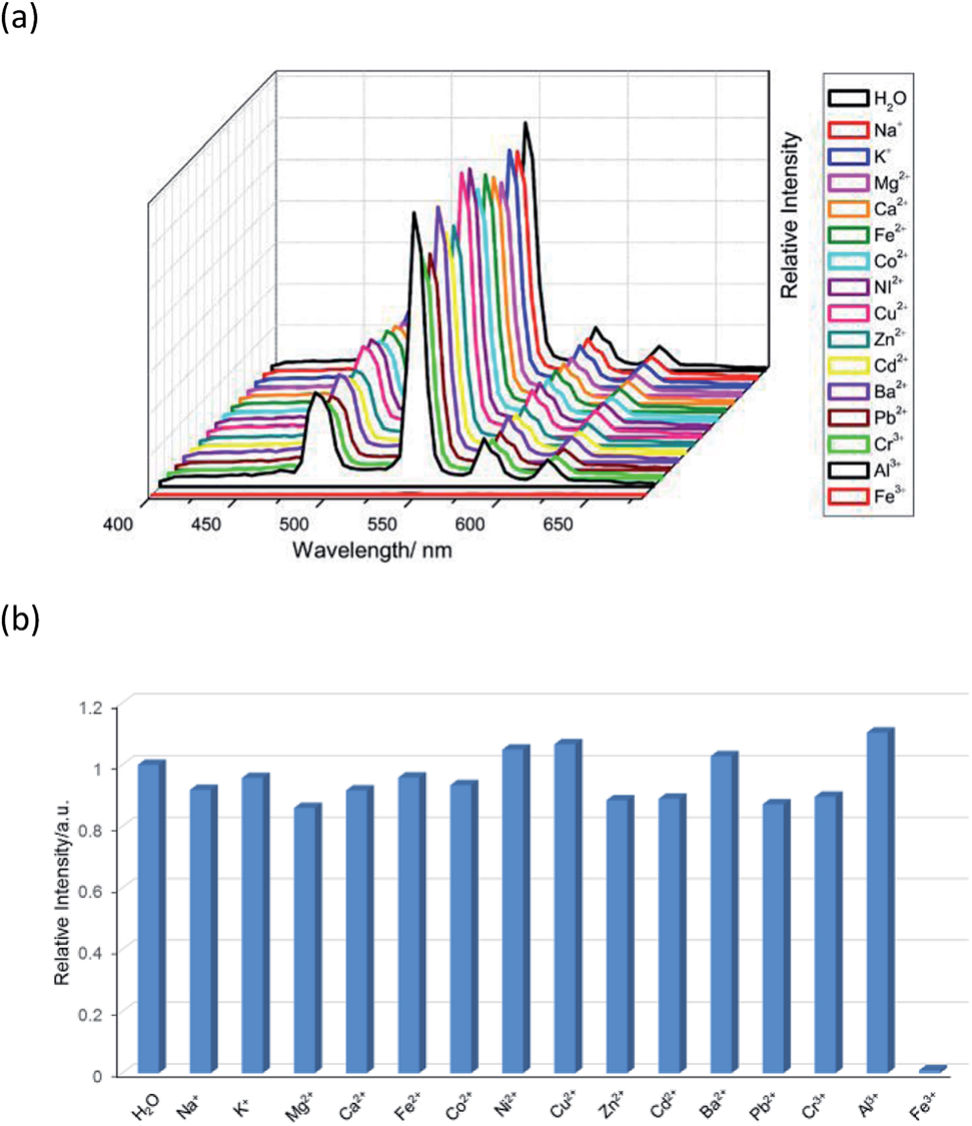
(a) The emission spectra and (b) relative intensities at 544 nm (^5^D_4_ → ^7^F_5_) for 2 which was dispersed in aqueous solutions of different metal ions upon excitation at 360 nm.

**Fig. 6:**
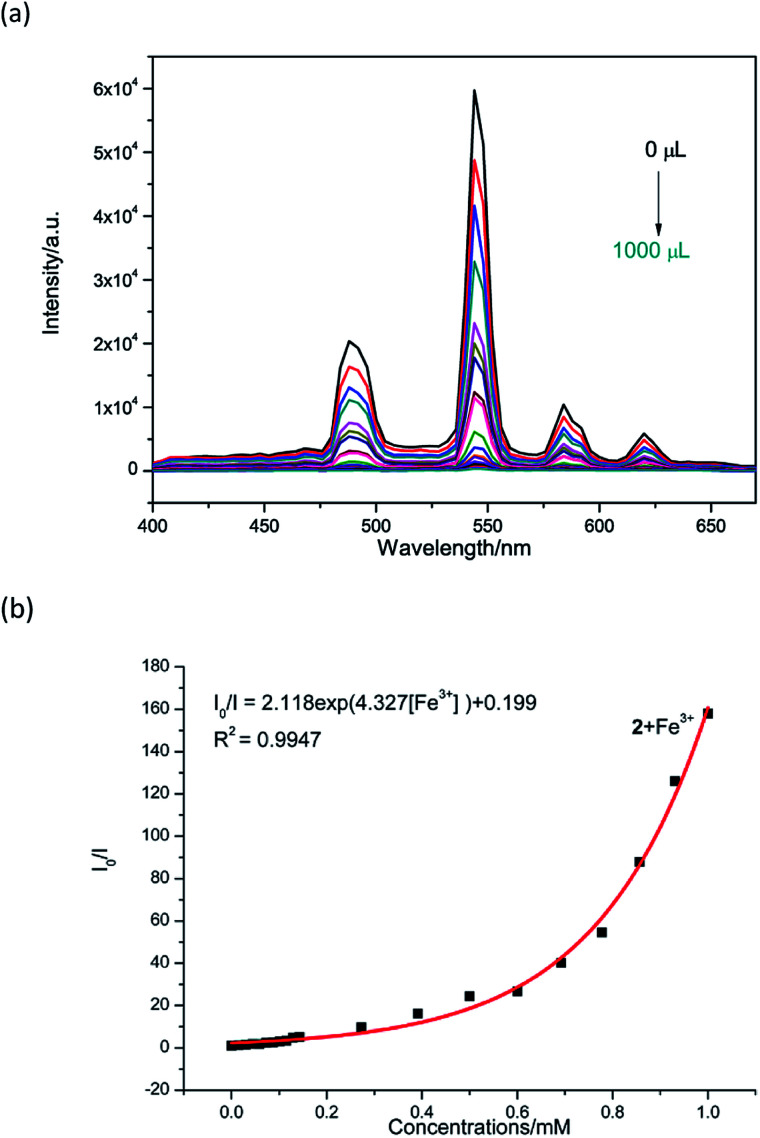
(a) Luminescent responses of a water suspension of 2 (2 mg mL^−1^) towards different concentrations of Fe^3+^ ions (3 × 10^−3^ M, 0–1000 μL). (b) Nonlinear Stern–Volmer (SV) plot for Fe^3+^ in the presence of a water suspension of 2 (2 mg/2 mL).

**Fig. 7:**
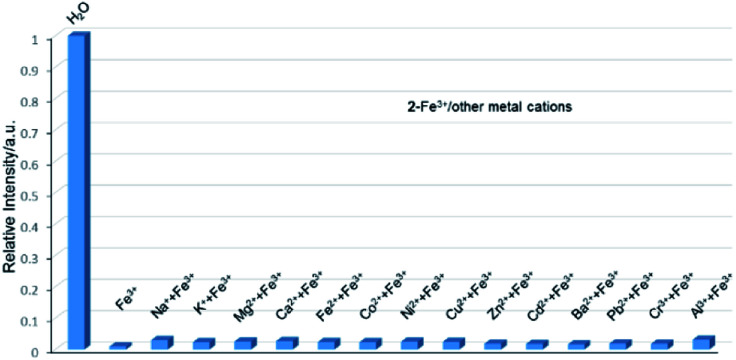
Relative luminescent intensities of the suspensions of 2 at 544 nm treated with Fe^3+^ in the presence of the other metal ions in water.

**Fig. 8:**
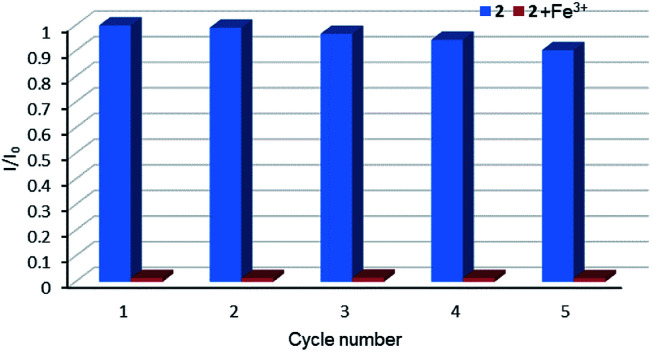
Relative luminescent intensity for ^5^D_4_ → ^7^F_5_ transition of suspension of 2 in the absence and presence of Fe^3+^ for each cyclic test.

Therefore, the resulting suspensions were sonicated for 30 minutes. Next, aqueous solutions of nitrate salts (200 μL, 10^−2^ M) of Na^+^, K^+^, Mg^2+^, Ca^2+^, Fe^2+^, Co^2+^, Ni^2+^, Cu^2+^, Zn^2+^, Cd^2+^, Pb^2+^, Al^3+^, Cr^3+^ and Fe^3+^ were added in the above suspensions for fluorescence testing. As shown in Fig. 5, the metal salts slightly altered the luminescent intensity of 2, whereas Fe^3+^ ion exhibited a significant influence on the luminescence of 2. Concentration-based studies were performed by adding different amount of aqueous solutions of Fe^3+^ ion into the suspensions of complex 2 (2 mg in 2 mL deionized water). As shown in Fig. 6, the luminescent intensities of 2 progressively decreased as the concentrations of Fe^3+^ ion increased. The luminescent intensities and the concentrations of Fe^3+^ ions show good linear relationships at low concentrations. The limit of detection (LOD) value is calculated to be 1 × 10^−5^ M by 3*δ*/*s*, where *δ* is the standard deviation of fluorescent test for 10 blank measurements and *s* is the slope of the calibration curve (Fig. S13b†).^12*i*,*j*^ In addition, the quenching effect can be explained by the Stern–Volmer equation: *I*_0_/*I* = *K*_SV_[*Q*] + 1, where *I*_0_ and *I* are the luminescent intensities before and after addition of the target analyte, respectively. *K*_SV_ is the Stern–Volmer quenching constant (M^−1^) and [*Q*] is the concentration of the analyte.^12*p*^ As can be seen from Fig. S13a,† the Stern–Volmer (SV) plot for Fe^3+^ ion displays nearly linear relationships at low concentration with *K*_SV_ value of 1.86 × 10^4^ M^−1^, which is comparable to those of reported fluorescent sensors based on Ln-complexes (Table S3†). The S–V plot deviates from linearity at high concentration (Fig. 6b), which may be attributed to both the occurrence of static and dynamic quenching. Nonlinear S–V curve of 2 can be fitted well by an exponential quenching equation, *I*_0_/*I* = 2.118 exp (4.327[Fe^3+^]) + 0.199. The possible interference experiments were carried out for complex 2. The selectivity of 2 towards Fe^3+^ ion over other metal cations was verified by adding Fe^3+^ ion (10^−2^ M, 0.2 mL) to the suspensions of 2 (2.4 mL) in which other competitive metal ions (10^−2^ M, 0.2 mL) were introduced. Then the changes of the emission intensities were recorded. No obvious changes were observed, which suggested that fluorescence detection of 2 could not be interfered by the presence of other cations. Competitive experiments indicated that 2 has great potential as a highly selective sensor for Fe^3+^ ion (Fig. 7). To study the recycling performance of the fluorescent sensor, Fe^3+^ ion (10^−2^ M, 0.2 mL) was introduced to the suspension of complex 2 (2 mL), then washed by water (after Fe^3+^ ion being detected) with five cycles of sensing experiments. As shown in Fig. 8, the fluorescent intensity of the samples did not drop significantly after five cycles. The resulting *K*_SV_, LOD value, high selectivity, stability and recyclable property reveal that complex 2 can be considered as a fluorescent sensor for Fe^3+^ ion in aqueous system.

As reported, the possible fluorescence quenching mechanism for 2 towards Fe^3+^ ion mainly rises from three aspects: (a) the collapse of the structure;^28^ (b) the weak interactions between Fe^3+^ ion and the methoxy group at the terminal of 2;^29^ (c) the energy transfer between 2 and Fe^3+^ ion.^30^ In order to understand which part was the main contributor, the PXRD, X-ray photoelectron spectroscopy (XPS) and UV-Vis absorption spectra of analytes were determined. By comparing the PXRD pattern (Fig. S15†) of 2 before and after the detection of Fe^3+^ ion, we found that no obvious changes occurred in this process, which indicated that the structure of 2 did not shatter. Depending on the result of the PXRD patterns, the fluorescence quenching mechanism of structural collapse can be ruled out. As shown in XPS spectrum of 2 (Fig. S14†), after the detection of Fe^3+^ ion, the O1s peak did not change, demonstrating the nonexistence of the weak interactions between Fe^3+^ ion and complex 2. However, as shown in Fig. S14,† the absorption band of Fe^3+^ ion from 250 nm to 400 nm remarkably overlaps the excitation band of 2 from 300 to 400 nm. Consequently, the solution of Fe^3+^ may absorb the energy of the excitation wavelength, which leads to the luminescence quenching.

### Detection of anions

**Fig. 9:**
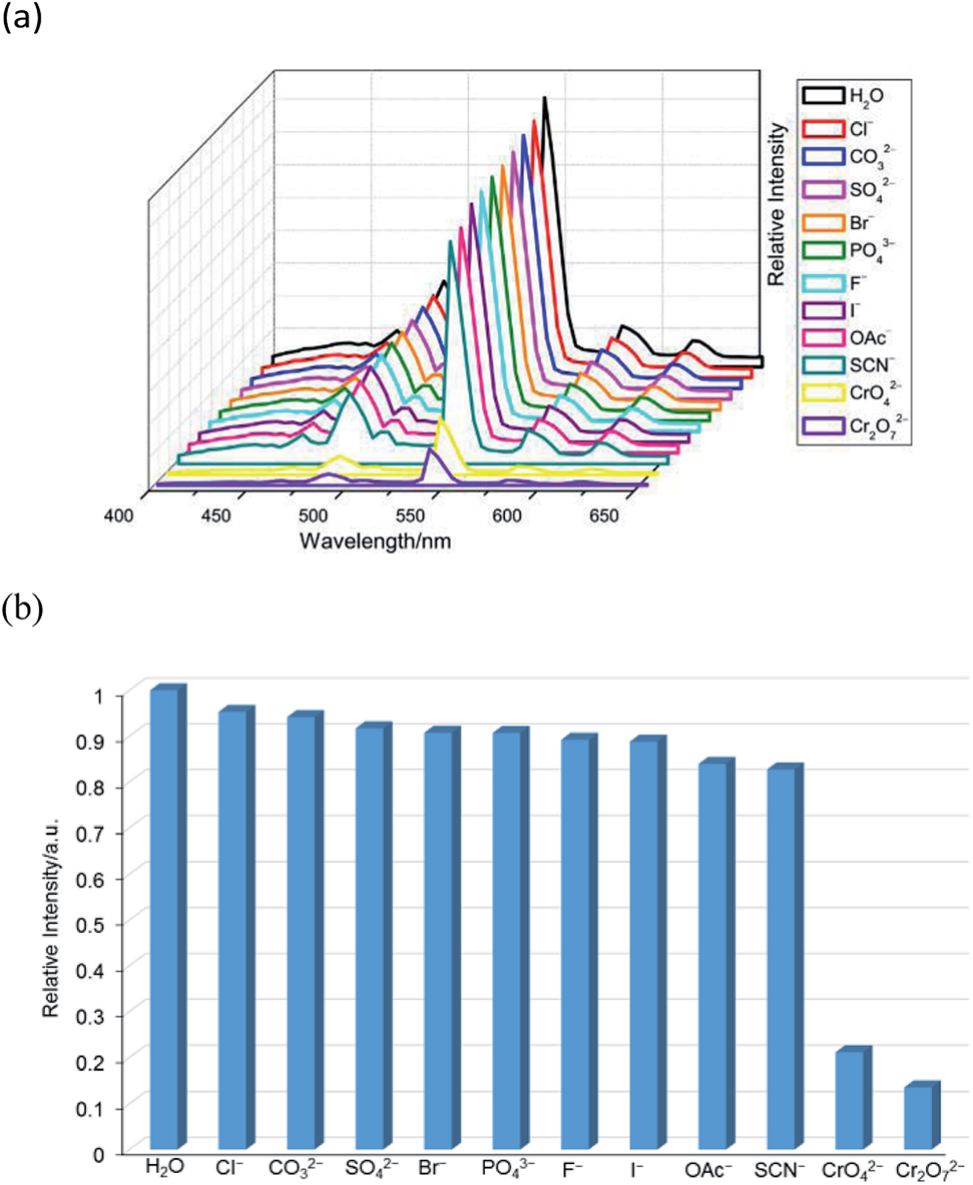
(a) The emission spectra and (b) relative intensities at 544 nm (^5^D_4_ → ^7^F_5_) for 2 dispersed in different anion aqueous solutions upon excitation at 360 nm.

**Fig. 10:**
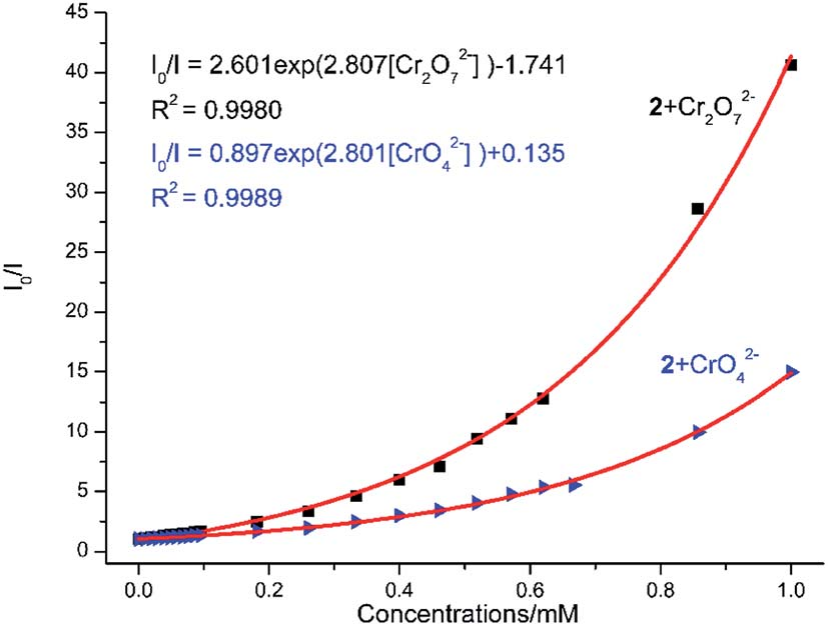
Nonlinear Stern–Volmer (SV) plot for CrO_4_^2−^/Cr_2_O_7_^2−^ in the presence of a water suspension of 2 (2 mg/2 mL).

**Fig. 11:**
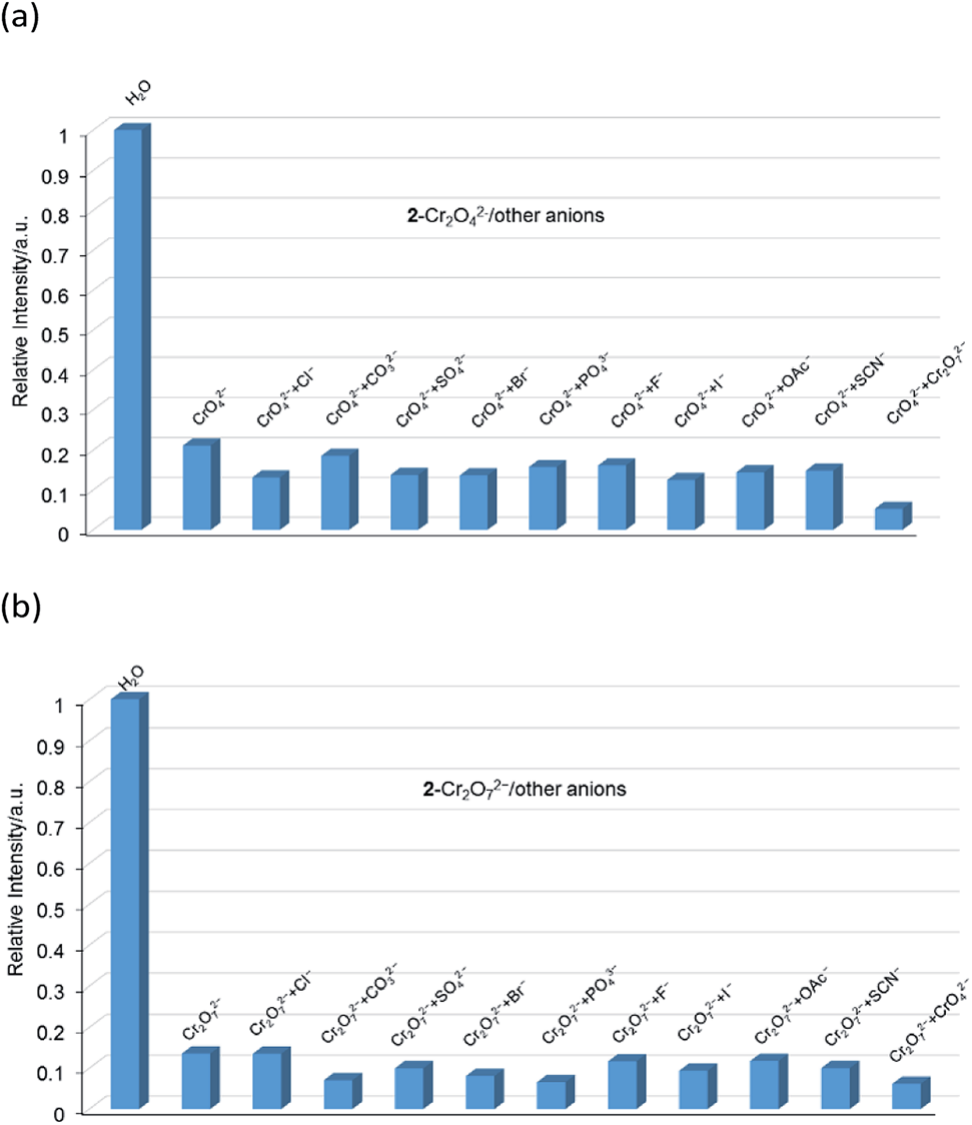
(a) Relative luminescent intensities of the suspension of 2 at 544 nm treated with CrO_4_^2−^ in the presence of the other anions in water. (b) Relative luminescent intensities of the suspension of 2 at 544 nm treated with Cr_2_O_7_^2−^ in the presence of the other anions in water.

**Fig. 12:**
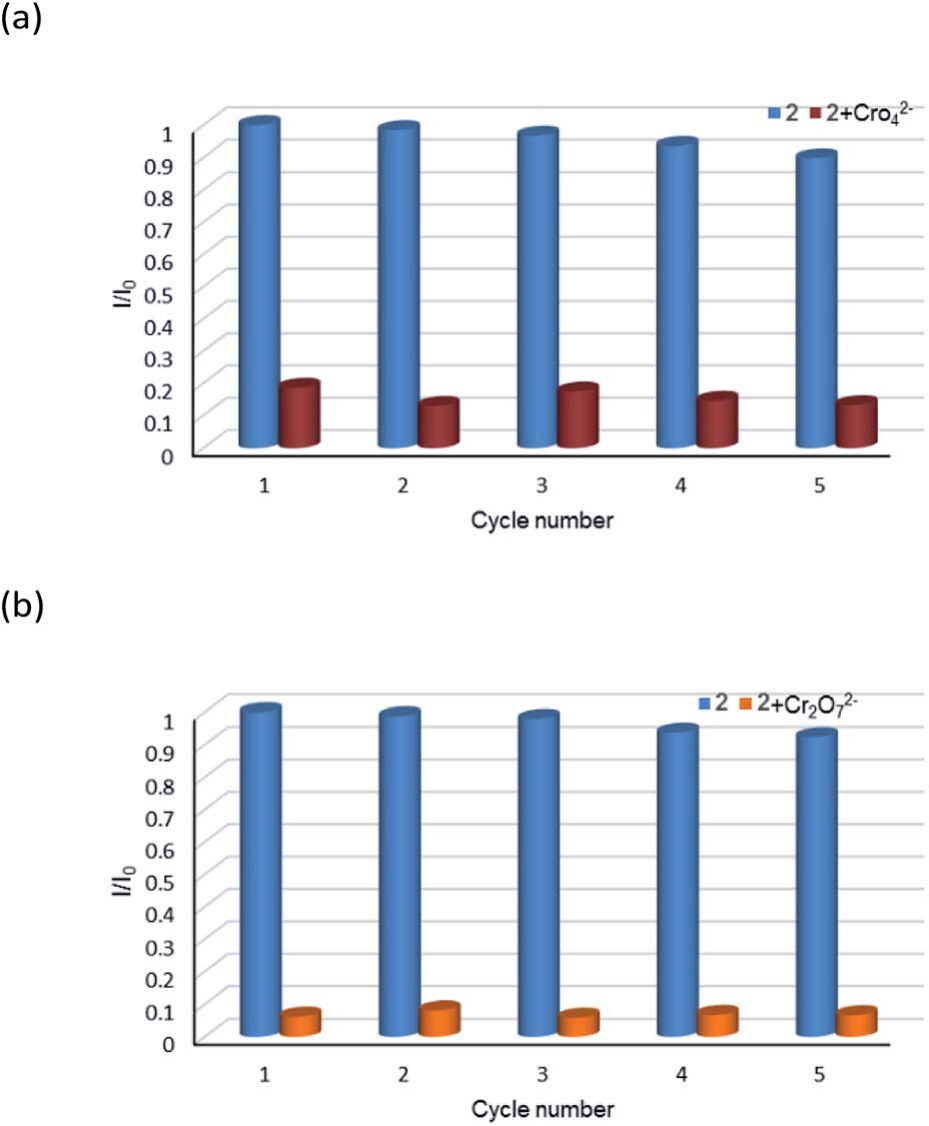
Relative luminescence intensity for ^5^D_4_ → ^7^F_5_ transition of a suspension of 2 in the absence and presence of CrO_4_^2−^ anions (a) and Cr_2_O_7_^2−^ anions (b) for each cyclic test.

The aqueous solutions (200 μL, 10^−2^ M) of common sodium salts Na_y_X (X = F^−^, Cl^−^, Br^−^, I^−^, NO_3_^−^, OAc^−^, SCN^−^, SO_4_^2−^, CO_3_^2−^, Cr_2_O_7_^2−^, CrO_4_^2−^, and PO_4_^3−^ were added in the suspensions of 2 (2 mg complex 2 in 2 mL water, and then being sonicated for 30 minutes) to investigate the luminescence quenching effects. An alluring finding is that CrO_4_^2−^ and Cr_2_O_7_^2−^ ions showed remarkable turnoff quenching effect on florescent intensities of 2, whereas other tested anions displayed minor effects on the luminescent intensity (Fig. 9). In order to further investigate the sensitivity of 2 toward CrO_4_^2−^ and Cr_2_O_7_^2−^, we implemented the titration experiments by adding aqueous solutions of CrO_4_^2−^ or Cr_2_O_7_^2−^ (2 × 10^−3^ M) to the suspension of complex 2 (2 mg in 2 mL water). The emission titration data were collected. As expected, the fluorescent intensity of 2 gradually decreased with the increase of the concentrations of CrO_4_^2−^ or Cr_2_O_7_^2−^ (Fig. S16†). Moreover, the S–V curve of 2 (Fig. 10) can be fitted well by an exponential quenching equation, *I*_0_/*I* = 0.897 exp(2.801[CrO_4_^2−^]) + 0.135 for CrO_4_^2−^ and *I*_0_/*I* = 2.601 exp(2.807[Cr_2_O_7_^2−^]) − 1.747 for Cr_2_O_7_^2−^. The S–V plot shows good linear relationships at low concentrations (Fig. S17a and S17b†), suggesting that both static and dynamic quenching may happen simultaneously. The quenching constants, *K*_SV_, are calculated to be 2.998 × 10^3^ M^−1^ (CrO_4_^2−^) and 7.44 × 10^3^ M^−1^ (Cr_2_O_7_^2−^) for 2 (Fig. S17a and S17b†). The LOD values calculated according to 3*δ*/*s* reach 5.2 × 10^−5^ M (CrO_4_^2−^) and 2.7 × 10^−5^ M (Cr_2_O_7_^2−^) for 2 (Fig. S17c and S17d†). Competitive experiments were carried out to study the interference of other ions on the fluorescent sensitivity for 2 towards CrO_4_^2−^ or Cr_2_O_7_^2−^ ions. The fluorescent intensity decreased significantly after adding CrO_4_^2−^ or Cr_2_O_7_^2−^ (0.2 mL, 10^−2^ M) into suspensions of 2 (2 mg in 2.2 mL water), in which other anions (0.2 mL, 10^−2^ M) were also added (Fig. 11). Moreover, recyclability experiments on the detection of CrO_4_^2−^ or Cr_2_O_7_^2−^ were performed. After five cycles of quenching experiments and regenerations, the quenching efficiency experiences only a minor decrease (Fig. 12). These results showed that complex 2 could be a potential candidate for sensitive and selective detections of CrO_4_^2−^ or Cr_2_O_7_^2−^ ions. A comparison of florescent sensing properties based on 2, Ln-MOF, and Ln-CP towards Fe^3+^, CrO_4_^2−^ or Cr_2_O_7_^2−^ ions is listed in Table S3.†^6*b*,12*f*–*o*,13*a*^

**Fig. 13:**
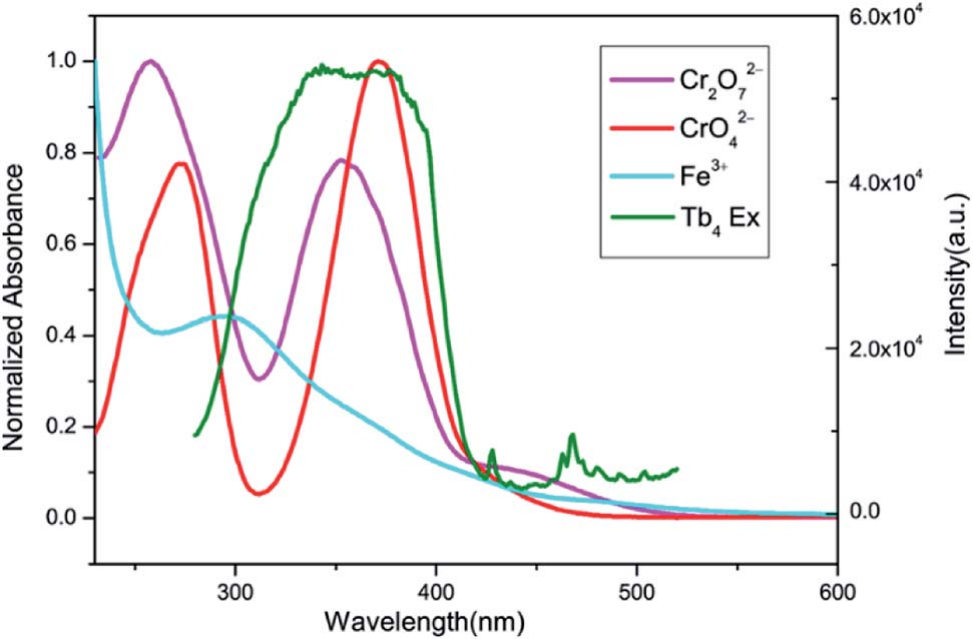
Spectral overlap between normalized absorption spectra of solutions of Fe^3+^, CrO_4_^2−^, Cr_2_O_7_^2−^ ions and excitation spectrum of 2 in water.

After CrO_4_^2−^ and Cr_2_O_7_^2−^ ions were detected, the PXRD patterns of 2 were measured. The obtained PXRD patterns still well correspond with the original data of 2, which demonstrate that the crystal structure of 2 is retained (Fig. S20†). On the other hand, the UV-Vis spectra of CrO_4_^2−^ and Cr_2_O_7_^2−^ ions in aqueous solution were determined. As shown in Fig. 13, the absorption bands of CrO_4_^2−^ in the range of 230–450 nm and Cr_2_O_7_^2−^ in the range of 275–475 nm display extensive overlap with the excitation band of 2. Therefore, luminescence quenching can be attributed to reducing the efficiency of the energy transfer from the ligand to the lanthanide ions, as the energy of the excitation light will be strongly absorbed by CrO_4_^2−^ or Cr_2_O_7_^2−^.^6*b*,13*e*^

### Detection of nitrobenzene derivatives

**Fig. 14:**
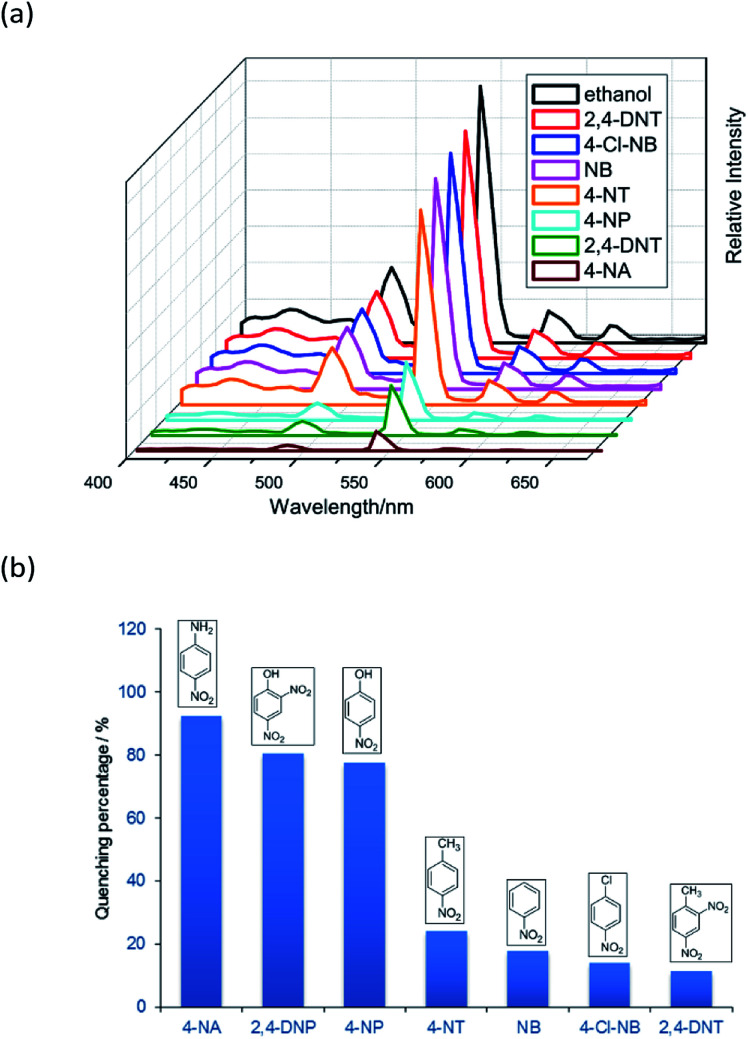
(a) The emission spectra and (b) luminescence quenching at 544 nm (^5^D_4_ → ^7^F_5_) for the suspensions of 2 in the presence of seven different ethanol solutions of NACs (10 μL, 10^−1^ M) upon excitation at 360 nm.

**Fig. 15:**
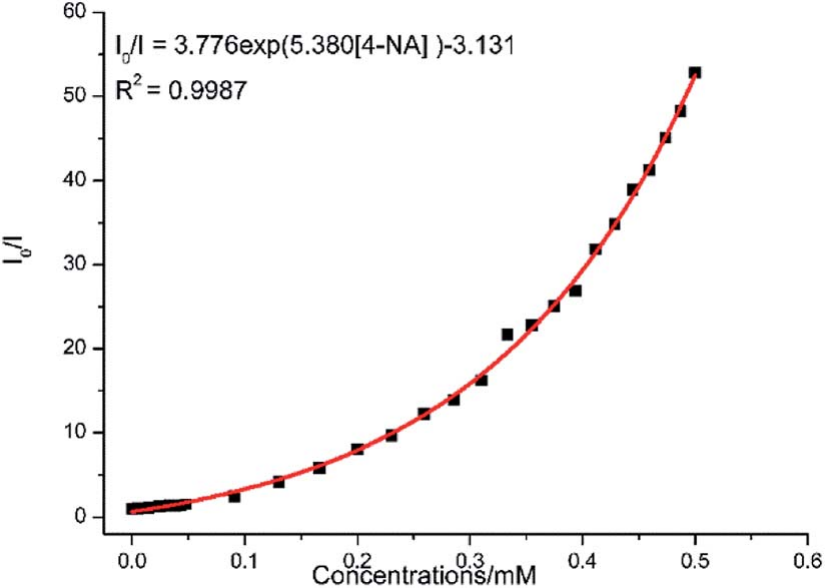
Nonlinear Stern–Volmer (SV) plot for 4-NA in the presence of an ethanol suspension of 2 (2 mg/2 mL).

**Fig. 16:**
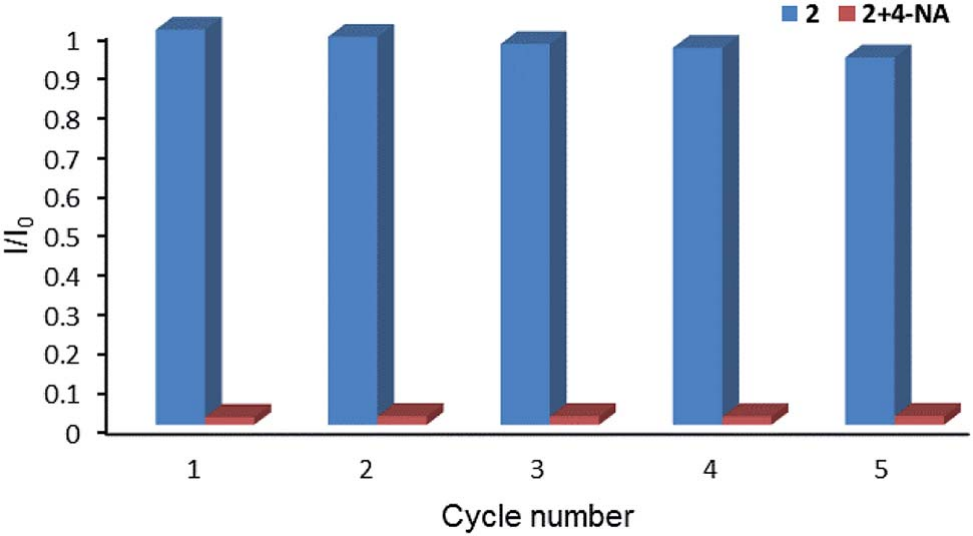
Relative luminescence intensity for ^5^D_4_ → ^7^F_5_ transition of ethanol suspension of 2 in the absence and presence of 4-NA for each cyclic test.

Complex 2 was dispersed in ethanol and then stirred for three days. It was filtered and dried. The PXRD of 2 (Fig. S20†) after being stirred for three days matched with that of the simulated data, which indicated that complex 2 was stable in ethanol solution. The stable nature of 2 in ethanol solution affords a prerequisite for the detection of nitroaromatics (NACs). Nitrobenzene (NB), 4-nitrophenol (4-NP), 4-nitroaniline (4-NA), 4-nitrotoluene (4-NT), 4-nitrochlorobenzene (4-Cl-NB), 2,4-dinitrotoluene (2,4-DNT) and 2,4-dinitrophenol (2,4-DNP) were selected to explore the potential of 2 as a chemosensor in ethanol solution. Firstly, complex 2 (2 mg) was homogeneously dispersed in a series of ethanol solution (2 mL) and sonicated for 30 minutes to form a uniform suspension. Then, different NACs (10 μL, 10^−1^ M) was added to the above-mentioned suspensions, respectively. Next, the fluorescent intensities of the resulting mixture were measured. As shown in Fig. 14, the order of quenching extend is as follows: 4-NA > 2,4-DNP > 4-NP > 4-NT > NB > 4-Cl-NB > 2,4-DNT. Concentration-dependent titration experiments of 4-NA were performed to test the influence of concentrations on fluorescent intensity. As the concentration of 4-NA increases, the fluorescent intensity gradually decreased (Fig. S18†). The liner fitting of the S–V plot for 4-NA at low concentrations (Fig. S19a†) gives *K*_SV_ value of 1.14 × 10^4^ M^−1^ (4-NA). Nonlinear concentration curve (Fig. 15) can be fitted well by an exponential quenching equation: *I*_0_/*I* = 3.776 exp(5.380[*Q*]) − 3.131 (*Q* = 4-NA). The detection limit value (Fig. S19b†) towards 4-NA was calculated by 3*δ*/*s*, generating 1.17 ppm (8.5 × 10^−6^ M) for 2. Recyclability experiments on the detection of 4-NA were performed. After five cycles of quenching experiments and regenerations, the quenching efficiency experiences only a minor decrease (Fig. 16). To our knowledge, few fluorescent complexes acted as sensors for 4-NA in ethanol system have been reported in the literature (Table S4†).^12*q*–*s*,13*b*^ Compared to luminescent sensors based on transition metal complexes, lanthanide complex 2 has the advantage of characteristic emissions and bright luminescent colour of Tb(iii) ion. No lanthanide clusters as sensors for 4-NA have been reported.

**Fig. 17:**
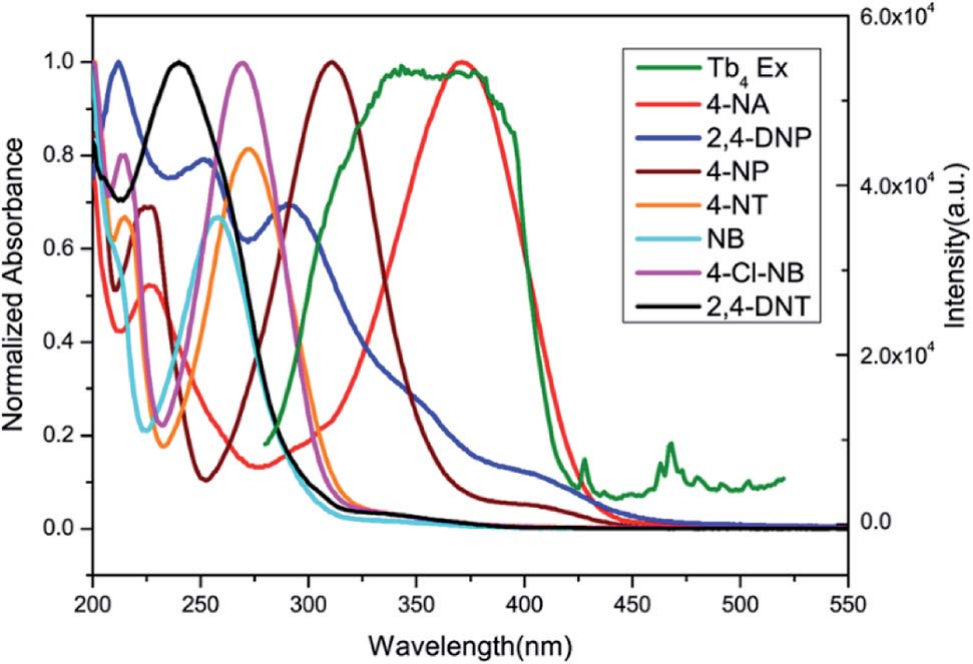
Spectral overlap between normalized absorption spectra of seven selected NACs and excitation spectra of 2 in ethanol solution.

As reported, the quenching mechanism of NACs sensors is ascribed to two factors: photo-induced electron transfer (PET) and resonance energy transfer (RET) or their cooperative effect. According to the molecular orbital theory, nitroaromatic compounds are good electron acceptors, due to the substitution of the electron-withdrawing nitro groups on the aromatic ring, which can stabilize the lowest unoccupied molecular orbital (LUMO) of the system *via* conjugation effect, ultimately leading to the complete or part quenching of luminescence-based sensor.^13*b*,31^ The electron transfer originated from the phenyl rings of the ligands of 2 to excellent electron donor nitrobenzene leads to luminescence quenching upon excitation. On the other hand, the UV-Vis results reveal that there are obvious overlaps between the absorption spectrum of NACs especially 4-NA and the emission spectrum of 2 (Fig. 17). This would permit energy transfer from 2 to NACs, thus further improving the luminescence quenching efficiency towards NACs.^30*b*^

## Conclusions

In summary, five tetranuclear lanthanide clusters have been synthesized by using Ln(NO_3_)_3_·6H_2_O (Ln = Gd, Tb, Dy, Ho, Er), pivalic acid and Schiff base ligand 2-(((2-hydroxy-3-methoxybenzyl)imino)methyl)-6-methoxyphenol (H_2_L) as starting materials under solvothermal method. Complexes 1–5 possess [Ln_4_O_6_] cores formed by the fusion of two phenoxide oxygen bridged two [Ln_2_O_2_] moieties. The magnetic properties of 1–5 were investigated. Antiferromagnetic interactions were determined for 1, 2, 4 and 5. Complex 3 displayed typical single molecule magnet behaviour. Considering its low detection limits and high quenching constant *K*_sv_, complex 2 may potentially be utilized as luminescence sensors for quantitative detection of 4-NA, Fe^3+^ and CrO_4_^2−^/Cr_2_O_7_^2−^ ions. Although the design of chemosensors is more based on academic or research interest, the exceptional stability of Tb_4_ clusters makes it applicable to extreme industrial application.

## Conflicts of interest

There are no conflicts to declare.

## Supplementary Material

RA-008-C8RA01485J-s001

RA-008-C8RA01485J-s002
