# Supplementary material for: A series of Ln4III clusters: Dy4 single molecule magnet and Tb4 multi-responsive luminescent sensor for Fe3+, CrO42−/Cr2O72− and 4-nitroaniline
Source: RSC Adv. 2018 Apr 3;8(23):12641–52. doi: 10.1039/c8ra01485j (PMC9079637; doi:10.1039/c8ra01485j)
Supplement: RA-008-C8RA01485J-s001 [file RA-008-C8RA01485J-s001.pdf]

Electronic Supplementary Material (ESI) for RSC Advances.
This journal is © The Royal Society of Chemistry 2018

A series of Ln^{III}_4 clusters: Dy_4 single molecule magnet and Tb_4 multi-responsive luminescent sensor for Fe^{3+}, $CrO_4^{2-}/Cr_2O_7^{2-}$ 4-nitroaniline

Yaru Qin, Yu Ge, Shasha Zhang, Hao Sun, Yu Jing, Yahong Li* and Wei Liu

College of Chemistry, Chemical Engineering and Materials Science, Soochow University, Suzhou 215123, China.
E-mail: liyahong@suda.edu.cn

Content

Fig. S1-S3 ^1H, ^{13}C NMR and IR spectra of H_2L......2

Table S1 Selected bond lengths and angles for **1**-**5**......3

Fig. S4 PXRD patterns for **1**-**5**......6

Fig. S5 IR spectra of **1**-**5**......7

Table S2 Results of Continuous Shape Measures (SHAPE) calculation......8

Fig. S6 The Curie-Weiss law fit of **1**......9

Fig. S7 Temperature dependence of the out-of-phase (χ'') ac susceptibility for **3**......9

Fig. S8 The excitation and emission spectra of H_2L and emission spectra of **1**-**5** in solid state......9

Fig. S9 Excitation spectrum and emission spectrum of **2** in solid state......10

Fig. S10-S11 PXRD patterns of **2** for pH and stability experiments......10

Fig. S12 SEM images and particle size distribution of **2** 11

Fig. S13 Plots of I_0/I-1 and fluorescence intensity of **2** versus low concentration of Fe^{3+}......11

Fig. S14 The XPS spectra of **2** and **2**-Fe^{3+}......12

Fig. S15 PXRD patterns for **2** after the detection of Fe^{3+}, CrO_4^{2-} and $Cr_2O_7^{2-}$......12

Fig. S16 Luminescent responses of **2** towards different concentrations of CrO_4^{2-} and $Cr_2O_7^{2-}$, respectively......12

Fig. S17 Plot of I_0/I-1 and fluorescence intensity of **2** versus low concentration of CrO_4^{2-} and $Cr_2O_7^{2-}$......13

Fig. S18 Luminescent responses of **2** towards different concentrations of 4-NA......13

Fig. S19 Plot of I_0/I-1 and fluorescence intensity of **2** for 4-NA in low concentration region......14

Fig. S20 PXRD patterns of **2** for free and 4-NA ethanol stability experiments...14

Table S3 Comparison of various Ln-complexes fluorescent sensors for Fe^{3+}, CrO_4^{2-} and $Cr_2O_7^{2-}$............................14

Table S4 Comparison of various complexes fluorescent sensors for 4-NA..15

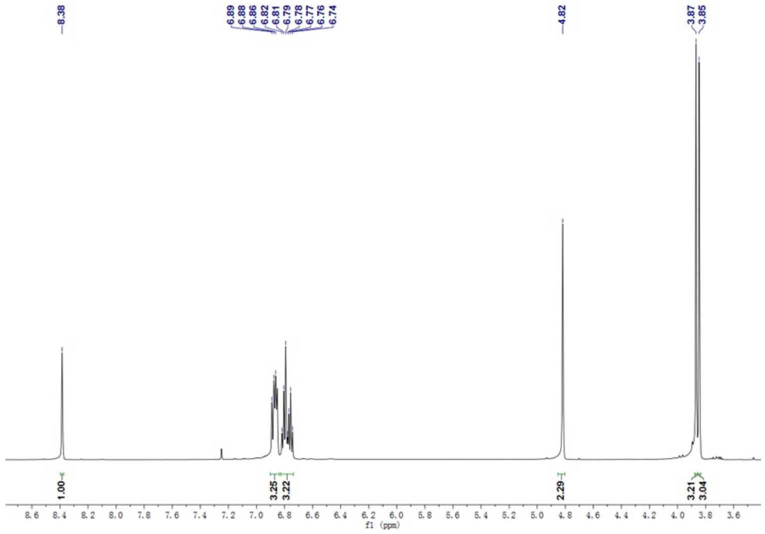

Fig. S1 ^1H NMR spectrum of H_2L.

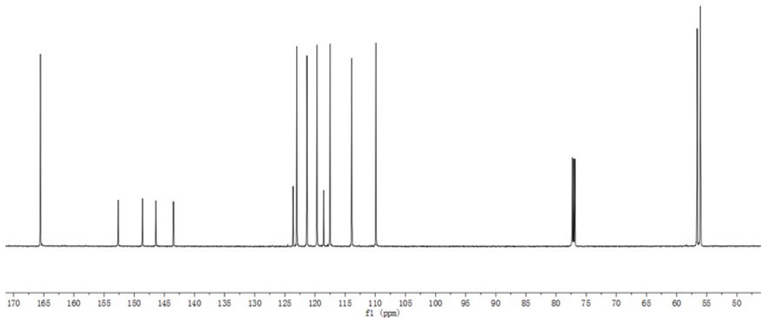

Fig. S2 ^{13}C NMR spectrum of H_2L.

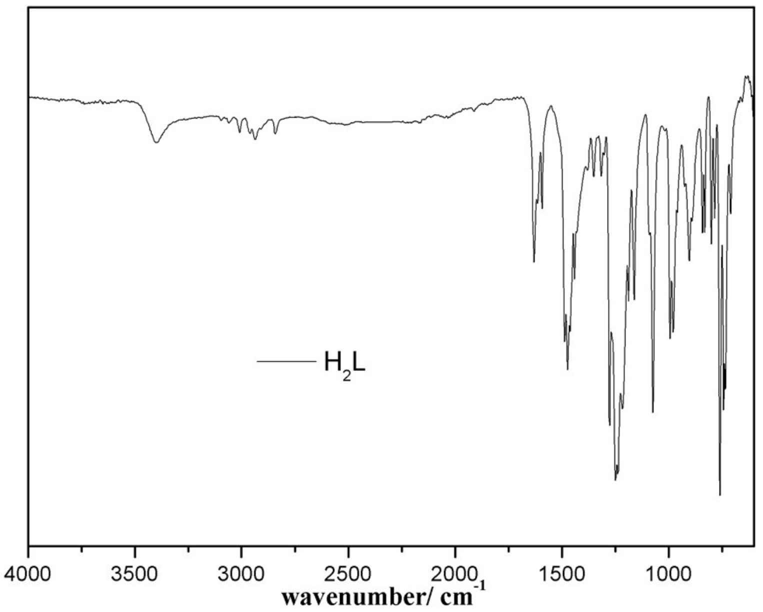

Fig. S3 FT-IR spectrum of H_2L.

Table S1 The selected bond length (Å) and angle (°) for **1**-**5**

**1**	**Length/Å**	**2**	**Length/Å**	**3**	**Length/Å**
Gd(1)-O(1)	2.310(3)	Tb(1)-O(1)	2.293(2)	Dy(1)-O(1)	2.293(2)
Gd(1)-O(3)	2.202(3)	Tb(1)-O(3)	2.181(2)	Dy(1)-O(3)	2.169(3)
Gd(1)-O(5)	2.451(3)	Tb(1)-O(5)	2.427(2)	Dy(1)-O(5)	2.405(2)
Gd(1)-O(6)	2.444(3)	Tb(1)-O(6)	2.435(2)	Dy(1)-O(6)	2.429(3)
Gd(1)-O(10)	2.368(3)	Tb(1)-O(10)	2.358(2)	Dy(1)-O(10)	2.355(3)
Gd(1)-O(11)	2.548(4)	Tb(1)-O(11)	2.533(3)	Dy(1)-O(11)	2.534(3)
Gd(1)-O(12)	2.478(4)	Tb(1)-O(12)	2.474(3)	Dy(1)-O(12)	2.455(3)
Gd(1-N(1)	2.509(4)	Tb(1)-N(1)	2.495(3)	Dy(1)-N(1)	2.491(3)
Gd(2)-O(1)	2.332(3)	Tb(2)-O(1)	2.314(2)	Dy(2)-O(1)	2.298(2)
Gd(2)-O(2)	2.586(3)	Tb(2)-O(2)	2.578(2)	Dy(2)-O(2)	2.575(3)
Gd(2)-O(5)	2.391(3)	Tb(2)-O(5)	2.379(2)	Dy(2)-O(5)	2.378(2)
Gd(2)-O(7)	2.305(3)	Tb(2)-O(7)	2.289(2)	Dy(2)-O(7)#1	2.303(2)
Gd(2)-O(7)#1	2.332(3)	Tb(2)-O(7)#1	2.312(2)	Dy(2)-O(7)	2.281(2)
Gd(2)-O(8)#1	2.634(4)	Tb(2)-O(8)#1	2.640(3)	Dy(2)-O(8)#1	2.635(3)
Gd(2)-O(9)	2.280(3)	Tb(2)-O(9)	2.258(2)	Dy(2)-O(9)	2.249(3)
Gd(2)-N(2)	2.479(5)	Tb(2)-N(2)	2.450(3)	Dy(2)-N(2)	2.443(3)
**4**	**Length/Å**	**4**	**Length/Å**	**4**	**Length/Å**
Ho(1)-O(1)	2.276(3)	Ho(1)-O(6)	2.408(3)	Ho(1)-O(12)	2.430(3)
Ho(1)-O(3)	2.167(3)	Ho(1)-O(10)	2.334(3)	Ho(1)-N(1)	2.472(4)
Ho(1)-O(5)	2.393(3)	Ho(1)-O(11)	2.519(3)	Ho(2)-O(1)	2.285(3)
Ho(2)-O(2)	2.568(3)	Ho(2)-O(5)	2.359(3)	Ho(2)-O(7)	2.268(3)
Ho(2)-O(7)#1	2.289(3)	Ho(2)-O(8)#1	2.618(3)	Ho(2)-O(9)	2.234(3)
Ho(2)-N(2)	2.429(4)				
**5**	**Length/Å**	**5**	**Length/Å**	**5**	**Length/Å**
Er(1)-O(1)	2.264(3)	Er(1)-O(10)	2.328(3)	Er(2)-O(1)	2.276(3)
Er(2)-O(7)#1	2.277(3)	Er(1)-O(3)	2.160(3)	Er(1)-O(11)	2.519(3)

Er(2)-O(2)	2.572(3)	Er(2)-O(8)#1	2.614(3)	Er(1)-O(5)	2.389(3)
Er(1)-O(12)	2.417(3)	Er(2)-O(5)	2.344(3)	Er(2)-O(9)	2.224(3)
Er(1)-O(6)	2.400(3)	Er(1)-N(1)	2.457(3)	Er(2)-O(7)	2.262(3)
Er(2)-N(2)	2.424(4)				
**1**	**Angle/°**	**2**	**Angle/°**	**3**	**Angle/°**
O(1)-Gd(1)-O(5)	73.48(11)	O(1)-Tb(1)-O(5)	73.05(8)	O(1)-Dy(1)-O(5)	72.91(8)
O(1)-Gd(1)-O(6)	138.17(12)	O(1)-Tb(1)-O(6)	138.35(8)	O(1)-Dy(1)-O(6)	138.57(9)
O(1)-Gd(1)-O(10)	84.17(12)	O(1)-Tb(1)-O(10)	84.30(9)	O(1)-Dy(1)-O(10)	84.34(9)
O(1)-Gd(1)-O(11)	134.50(12)	O(1)-Tb(1)-O(11)	134.22(9)	O(1)-Dy(1)-O(11)	134.32(9)
O(1)-Gd(1)-O(12)	87.23(12)	O(1)-Tb(1)-O(12)	86.97(9)	O(1)-Dy(1)-O(12)	87.05(9)
O(1)-Gd(1)-N(1)	75.32(13)	O(1)-Tb(1)-N(1)	75.49(9)	O(1)-Dy(1)-N(1)	75.46(10)
O(3)-Gd(1)-O(1)	127.73(12)	O(3)-Tb(1)-O(1)	128.40(9)	O(3)-Dy(1)-O(1)	127.96(10)
O(3)-Gd(1)-O(5)	146.01(12)	O(3)-Tb(1)-O(5)	146.15(9)	O(3)-Dy(1)-O(5)	146.22(9)
O(3)-Gd(1)-O(6)	85.60(12)	O(3)-Tb(1)-O(6)	84.91(9)	O(3)-Dy(1)-O(6)	85.07(9)
O(3)-Gd(1)-O(10)	79.87(13)	O(3)-Tb(1)-O(10)	80.12(9)	O(3)-Dy(1)-O(10)	79.57(9)
O(3)-Gd(1)-O(11)	76.43(13)	O(3)-Tb(1)-O(11)	75.97(9)	O(3)-Dy(1)-O(11)	76.19(10)
O(3)-Gd(1)-O(12)	122.70(13)	O(3)-Tb(1)-O(12)	122.75(9)	O(3)-Dy(1)-O(12)	123.41(10)
O(3)-Gd(1)-N(1)	72.92(13)	O(3)-Tb(1)-N(1)	73.50(9)	O(3)-Dy(1)-N(1)	73.68(10)
O(5)-Gd(1)-O(11)	108.61(12)	O(5)-Tb(1)-O(6)	66.47(8)	O(5)-Dy(1)-O(6)	66.78(8)
O(5)-Gd(1)-O(12)	79.59(12)	O(5)-Tb(1)-O(11)	108.89(9)	O(5)-Dy(1)-O(11)	109.29(9)
O(5)-Gd(1)-N(1)	140.90(12)	O(5)-Tb(1)-O(12)	79.06(9)	O(5)-Dy(1)-O(12)	78.73(9)
O(6)-Gd(1)-O(5)	65.86(11)	O(5)-Tb(1)-N(1)	140.25(9)	O(5)-Dy(1)-N(1)	139.97(9)
O(6)-Gd(1)-O(11)	70.75(12)	O(6)-Tb(1)-O(11)	70.90(9)	O(6)-Dy(1)-O(11)	70.75(9)
O(6)-Gd(1)-O(12)	94.58(13)	O(6)-Tb(1)-O(12)	94.52(9)	O(6)-Dy(1)-O(12)	94.05(10)
O(6)-Gd(1)-N(1)	145.51(13)	O(6)-Tb(1)-N(1)	145.02(9)	O(6)-Dy(1)-N(1)	144.74(10)
O(10)-Gd(1)-O(5)	76.27(12)	O(10)-Tb(1)-O(5)	76.35(8)	O(10)-Dy(1)-O(5)	76.52(9)
O(10)-Gd(1)-O(6)	77.31(12)	O(10)-Tb(1)-O(6)	77.25(9)	O(10)-Dy(1)-O(6)	77.57(9)
O(10)-Gd(1)-O(11)	141.26(12)	O(10)-Tb(1)-O(11)	141.42(9)	O(10)-Dy(1)-O(11)	141.31(9)
O(10)-Gd(1)-O(12)	155.79(13)	O(10)-Tb(1)-O(12)	155.35(9)	O(10)-Dy(1)-O(12)	155.21(9)
O(10)-Gd(1)-N(1)	123.11(13)	O(10)-Tb(1)-N(1)	123.88(9)	O(10)-Dy(1)-N(1)	123.94(10)
O(12)-Gd(1)-O(11)	50.58(12)	O(12)-Tb(1)-O(11)	50.74(9)	O(12)-Dy(1)-O(11)	51.01(9)
O(12)-Gd(1)-N(1)	75.94(13)	O(12)-Tb(1)-N(1)	75.59(9)	O(12)-Dy(1)-N(1)	75.73(10)
N(1)-Gd(1)-O(11)	78.03(13)	N(1)-Tb(1)-O(11)	77.25(9)	N(1)-Dy(1)-O(11)	76.95(10)
O(1)-Gd(2)-O(2)	64.51(11)	O(1)-Tb(2)-O(2)	64.85(8)	O(1)-Dy(2)-O(2)	65.13(9)
O(1)-Gd(2)-O(5)	74.22(11)	O(1)-Tb(2)-O(5)	73.58(8)	O(1)-Dy(2)-O(5)	73.33(8)
O(1)-Gd(2)-O(7)#1	89.14(12)	O(1)-Tb(2)-O(8)#1	76.58(8)	O(1)-Dy(2)-O(7)#1	88.98(9)
O(1)-Gd(2)-O(8)#1	76.69(11)	O(1)-Tb(2)-N(2)	150.82(9)	O(1)-Dy(2)-O(8)#1	76.44(9)
O(1)-Gd(2)-N(2)	151.17(13)	O(2)-Tb(2)-O(8)#1	120.96(8)	O(1)-Dy(2)-N(2)	150.93(10)
O(2)-Gd(2)-O(8)#1	120.64(11)	O(5)-Tb(2)-O(2)	126.99(8)	O(2)-Dy(2)-O(8)#1	121.43(9)
O(5)-Gd(2)-O(2)	127.31(11)	O(5)-Tb(2)-O(8)#1	76.50(8)	O(5)-Dy(2)-O(2)	126.88(9)
O(5)-Gd(2)-O(8)#1	76.77(11)	O(5)-Tb(2)-N(2)	77.27(9)	O(5)-Dy(2)-O(8)#1	76.11(8)
O(5)-Gd(2)-N(2)	77.00(13)	O(7)#1-Tb(2)-O(1)	89.15(8)	O(5)-Dy(2)-N(2)	77.62(10)
O(7)-Gd(2)-O(1)	138.07(12)	O(7)-Tb(2)-O(1)	138.08(8)	O(7)-Dy(2)-O(1)	138.07(9)
O(7)-Gd(2)-O(2)	75.87(12)	O(7)#1-Tb(2)-O(2)	74.22(9)	O(7)#1-Dy(2)-O(2)	74.38(9)

O(7)#1-Gd(2)-O(2)	73.95(12)	O(7)-Tb(2)-O(2)	75.73(9)	O(7)-Dy(2)-O(2)	75.52(9)
O(7)-Gd(2)-O(5)	146.00(12)	O(7)-Tb(2)-O(5)	146.76(8)	O(7)-Dy(2)-O(5)	147.05(9)
O(7)#1-Gd(2)-O(5)	138.09(11)	O(7)#1-Tb(2)-O(5)	137.70(8)	O(7)#1-Dy(2)-O(5)	137.43(9)
O(7)-Gd(2)-O(7)#1	66.35(14)	O(7)-Tb(2)-O(7)#1	66.09(10)	O(7)-Dy(2)-O(7)#1	66.22(10)
O(7)#1-Gd(2)-O(8)#1	61.90(11)	O(7)#1-Tb(2)-O(8)#1	61.85(8)	O(7)#1-Dy(2)-O(8)#1	62.03(8)
O(7)-Gd(2)-O(8)#1	115.29(11)	O(7)-Tb(2)-O(8)#1	115.03(8)	O(7)-Dy(2)-O(8)#1	115.26(9)
O(7)-Gd(2)-N(2)	69.97(14)	O(7)#1-Tb(2)-N(2)	113.93(9)	O(7)-Dy(2)-N(2)	70.37(10)
O(7)#1-Gd(2)-N(2)	113.92(13)	O(7)-Tb(2)-N(2)	70.41(9)	O(7)#1-Dy(2)-N(2)	113.84(10)
O(9)-Gd(2)-O(1)	92.28(12)	O(9)-Tb(2)-O(1)	92.48(9)	O(9)-Dy(2)-O(1)	92.89(9)
O(9)-Gd(2)-O(2)	71.67(12)	O(9)-Tb(2)-O(2)	71.79(9)	O(9)-Dy(2)-O(2)	71.77(9)
O(9)-Gd(2)-O(5)	79.03(12)	O(9)-Tb(2)-O(5)	78.92(9)	O(9)-Dy(2)-O(5)	79.06(9)
O(9)-Gd(2)-O(7)	87.72(12)	O(9)-Tb(2)-O(7)	88.16(9)	O(9)-Dy(2)-O(7)	87.96(9)
O(9)-Gd(2)-O(7)#1	141.02(12)	O(9)-Tb(2)-O(7)#1	141.45(9)	O(9)-Dy(2)-O(7)#1	141.57(9)
O(9)-Gd(2)-O(8)#1	155.34(12)	O(9)-Tb(2)-O(8)#1	155.05(8)	O(9)-Dy(2)-O(8)#1	154.87(9)
O(9)-Gd(2)-N(2)	80.60(14)	O(9)-Tb(2)-N(2)	80.50(10)	O(9)-Dy(2)-N(2)	80.36(11)
N(2)-Gd(2)-O(2)	136.43(13)	N(2)-Tb(2)-O(2)	136.50(9)	N(2)-Dy(2)-O(2)	136.27(10)
N(2)-Gd(2)-O(8)#1	98.39(13)	N(2)-Tb(2)-O(8)#1	98.07(10)	N(2)-Dy(2)-O(8)#1	97.86(10)
**4**	**Angle/°**	**4**	**Angle/°**	**4**	**Angle/°**
O(1)-Ho(1)-O(5)	72.99(9)	O(1)-Ho(1)-O(6)	138.9(10)	O(1)-Ho(1)-O(10)	84.35(10)
O(1)-Ho(1)-O(11)	134.23(10)	O(1)-Ho(1)-O(5)	72.99(9)	O(1)-Ho(1)-O(6)	138.90(10)
O(1)-Ho(1)-O(12)	86.77(11)	O(1)-Ho(1)-N(1)	75.53(11)	O(3)-Ho(1)-O(1)	127.85(11)
O(3)-Ho(1)-O(5)	145.96(11)	O(3)-Ho(1)-O(6)	84.75(11)	O(3)-Ho(1)-O(10)	79.20(11)
O(3)-Ho(1)-O(11)	76.43(11)	O(3)-Ho(1)-O(12)	123.93(11)	O(3)-Ho(1)-N(1)	73.87(11)
O(5)-Ho(1)-O(6)	67.02(9)	O(5)-Ho(1)-O(11)	109.35(10)	O(5)-Ho(1)-O(12)	78.63(10)
O(5)-Ho(1)-N(1)	140.03(11)	O(6)-Ho(1)-O(11)	70.66(10)	O(6)-Ho(1)-O(12)	94.25(11)
O(6)-Ho(1)-N(1)	144.34(11)	O(10)-Ho(1)-O(5)	76.59(10)	O(10)-Ho(1)-O(6)	77.73(11)
O(10)-Ho(1)-O(11)	141.39(11)	O(10)-Ho(1)-O(12)	155.15(11)	O(10)-Ho(1)-N(1)	123.99(11)
O(12)-Ho(1)-O(11)	51.27(11)	O(12)-Ho(1)-N(1)	75.60(12)	N(1)-Ho(1)-O(11)	76.69(11)
O(1)-Ho(2)-O(2)	65.38(10)	O(1)-Ho(2)-O(5)	73.48(10)	O(1)-Ho(2)-O(7)#1	88.39(10)
O(1)-Ho(2)-O(8)#1	76.51(10)	O(1)-Ho(2)-N(2)	150.76(11)	O(2)-Ho(2)-O(8)#1	122.11(10)
O(5)-Ho(2)-O(2)	127.19(10)	O(5)-Ho(2)-O(8)#1	75.50(10)	O(5)-Ho(2)-N(2)	77.31(11)
O(7)-Ho(2)-O(1)	137.84(10)	O(7)-Ho(2)-O(2)	75.12(10)	O(7)#1-Ho(2)-O(2)	74.12(10)
O(7)-Ho(2)-O(5)	147.18(10)	O(7)#1-Ho(2)-O(5)	137.19(10)	O(7)-Ho(2)-O(7)#1	66.46(12)
O(7)#1-Ho(2)-O(8)#1	62.50(10)	O(7)-Ho(2)-O(8)#1	115.54(10)	O(7)-Ho(2)-N(2)	70.75(12)
O(7)#1-Ho(2)-N(2)	114.66(12)	O(9)-Ho(2)-O(1)	92.94(10)	O(9)-Ho(2)-O(2)	71.54(10)
O(9)-Ho(2)-O(7)	87.94(10)	O(9)-Ho(2)-O(7)#1	141.40(10)	O(9)-Ho(2)-O(8)#1	154.52(10)
O(9)-Ho(2)-N(2)	80.19(12)	N(2)-Ho(2)-O(2)	135.99(12)	N(2)-Ho(2)-O(8)#1	97.64(12)
**5**	**Angle/°**	**5**	**Angle/°**	**5**	**Angle/°**
O(1)-Er(1)-O(5)	72.76(9)	O(1)-Er(1)-O(6)	138.85(9)	O(1)-Er(1)-O(10)	84.24(10)

O(1)-Er(1)-O(11)	134.56(10)	O(1)-Er(1)-O(12)	86.88(10)	O(1)-Er(1)-N(1)	75.87(10)
O(3)-Er(1)-O(1)	128.17(10)	O(3)-Er(1)-O(5)	145.47(10)	O(3)-Er(1)-O(6)	84.16(10)
O(3)-Er(1)-O(10)	78.79(10)	O(3)-Er(1)-O(11)	76.26(10)	O(3)-Er(1)-O(12)	124.30(10)
O(3)-Er(1)-N(1)	74.47(11)	O(5)-Er(1)-O(6)	67.24(9)	O(5)-Er(1)-O(11)	109.70(10)
O(5)-Er(1)-O(12)	78.55(10)	O(5)-Er(1)-N(1)	139.93(10)	O(6)-Er(1)-O(11)	70.65(10)
O(6)-Er(1)-O(12)	94.25(11)	O(6)-Er(1)-N(1)	144.04(10)	O(10)-Er(1)-O(5)	76.59(10)
O(10)-Er(1)-O(6)	77.67(10)	O(10)-Er(1)-O(11)	141.18(10)	O(10)-Er(1)-O(12)	155.08(10)
O(10)-Er(1)-N(1)	124.31(11)	O(12)-Er(1)-O(11)	51.57(10)	O(12)-Er(1)-N(1)	75.43(11)
N(1)-Er(1)-O(11)	76.30(11)	O(1)-Er(2)-O(2)	65.43(9)	O(1)-Er(2)-O(5)	73.41(9)
O(1)-Er(2)-O(7)#1	88.60(10)	O(1)-Er(2)-O(8)#1	76.82(9)	O(1)-Er(2)-N(2)	151.00(11)
O(2)-Er(2)-O(8)#1	122.45(9)	O(5)-Er(2)-O(2)	127.27(9)	O(5)-Er(2)-O(8)#1	75.27(9)
O(5)-Er(2)-N(2)	77.63(11)	O(7)-Er(2)-O(1)	137.73(10)	O(7)-Er(2)-O(2)	74.96(10)
O(7)#1-Er(2)-O(2)	74.19(10)	O(7)-Er(2)-O(5)	147.38(10)	O(7)#1-Er(2)-O(5)	137.14(9)
O(7)-Er(2)-O(7)#1	66.24(12)	O(7)#1-Er(2)-O(8)#1	62.70(9)	O(7)-Er(2)-O(8)#1	115.46(9)
O(7)-Er(2)-N(2)	70.62(11)	O(7)#1-Er(2)-N(2)	114.29(11)	O(9)-Er(2)-O(1)	92.77(10)
O(9)-Er(2)-O(2)	71.29(10)	O(9)-Er(2)-O(5)	79.56(10)	O(9)-Er(2)-O(7)	87.92(10)
O(9)-Er(2)-O(7)#1	141.19(10)	O(9)-Er(2)-O(8)#1	154.58(10)	O(9)-Er(2)-N(2)	80.46(11)
N(2)-Er(2)-O(2)	135.78(11)	N(2)-Er(2)-O(8)#1	97.37(11)		

Symmetry transformation: #1 -X, 2-Y, 1-Z for **1**, #1 2-X, -Y, 1-Z for **2**-**5**

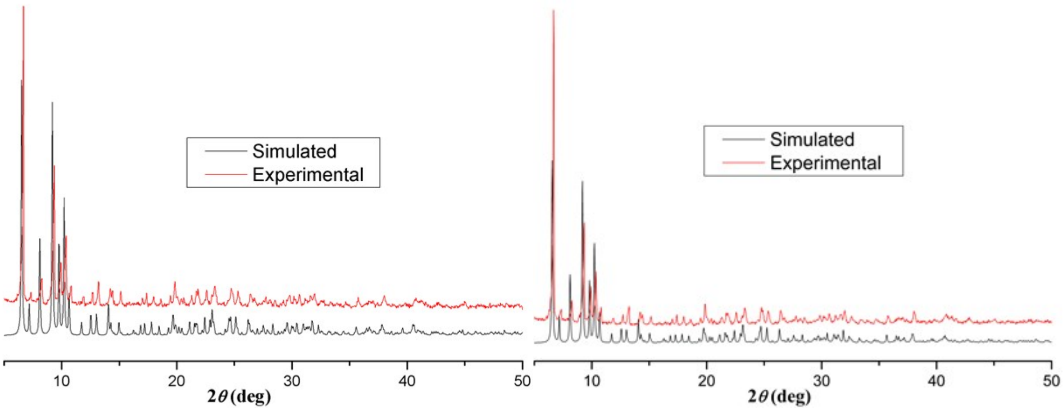

(a) (b)

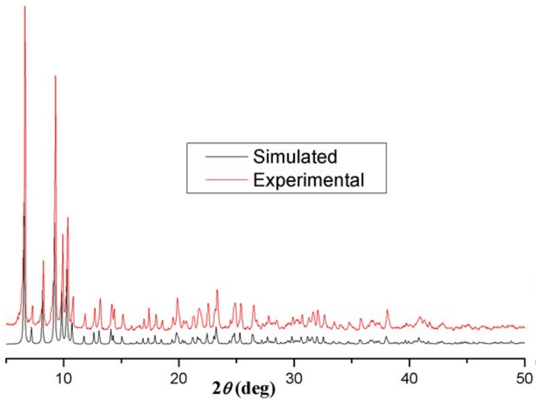

(c)

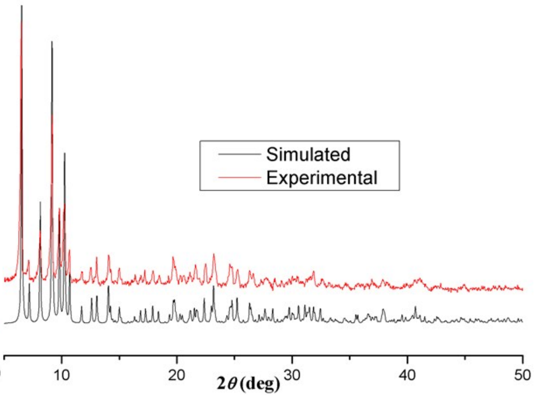

(d)

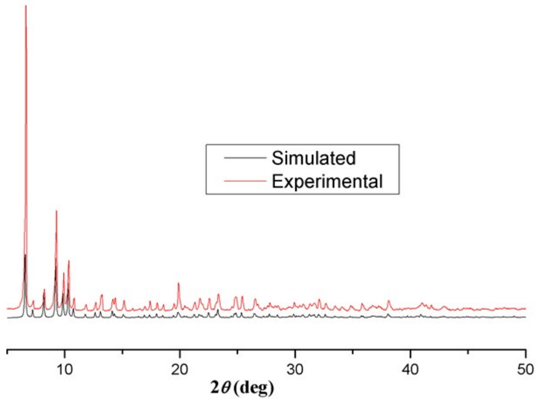

(e)

Fig. S4 PXRD patterns of **1**(a), **2**(b), **3**(c), **4**(d), **5**(e).

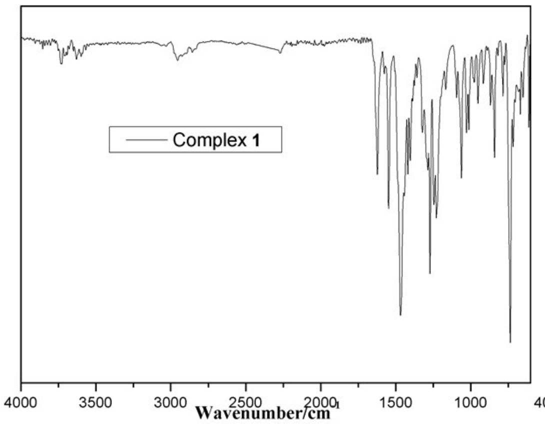

(a)

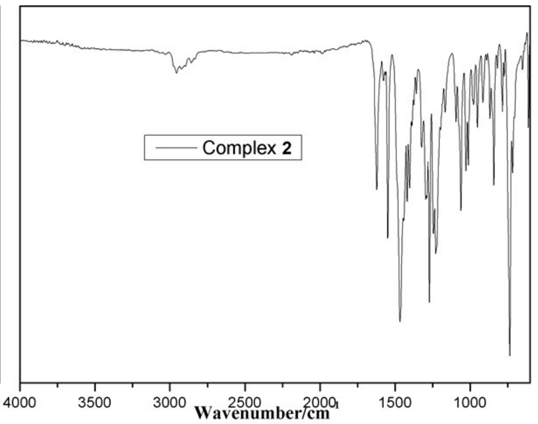

(b)

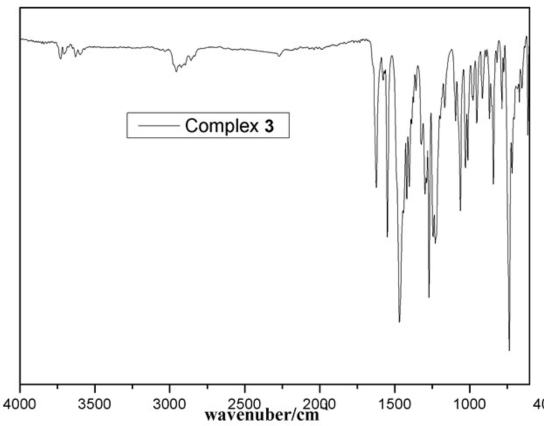

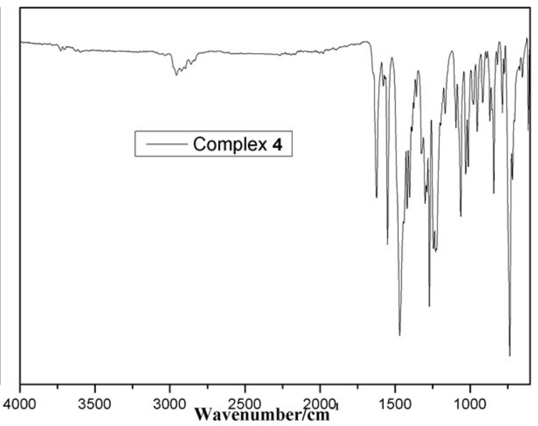

(c) (d)

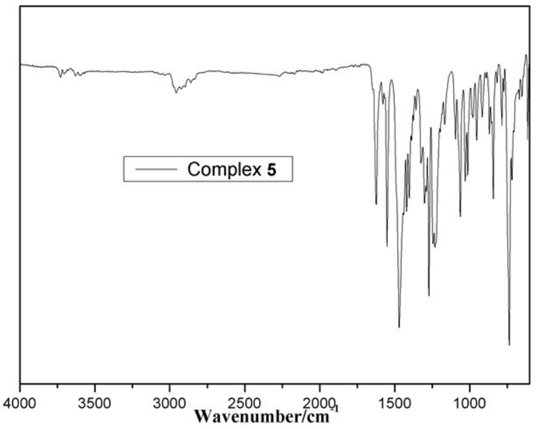

(e)

Fig. S5 IR spectra of **1**(a), **2**(b), **3**(c), **4**(d), **5**(e).

Table S2. Agreement factor between the coordination polyhedron of the Ln^{III} and the various ideal polyhedral calculated by the SHAPE program

	HBPY-8	CU-8	SAPR-8	TDD-8	JGBF-8	JBTPR-8	BTPR-8	JSD-8	TT-8
Gd1	14.779,	8.898,	1.686,	2.836,	14.348,	2.716,	1.948	5.147	9.501,
Gd2,	14.482,	11.904,	3.123,	2.877,	11.858,	2.745,	2.428	4.685	12.131
Tb1	14.941,	8.967,	1.635,	2.787,	14.521,	2.674,	1.858	5.070	9.572
Tb2	14.587,	11.972,	3.095,	2.913,	11.953,	2.721,	2.435	4.625	12.156
Dy1,	15.029,	9.008,	1.635,	2.709,	14.552,	2.604,	1.787	4.952	9.590
Dy2,	14.735,	12.056,	3.038,	2.904,	12.026,	2.668,	2.416	4.549	12.224
Ho1	15.177,	9.091,	1.567,	2.688,	14.556,	2.534,	1.741,	4.917	9.670
Ho2,	14.872,	12.189,	2.967,	2.924,	11.996,	2.572,	2.313	4.515	12.306
Er1	15.298,	9.127,	1.525,	2.645,	14.588,	2.447,	1.685	4.823	9.686
Er2	14.918	12.242	2.990	2.925	12.031	2.566	2.323	4.478	12.406

1 HBPY-8	Hexagonal bipyramid	D6h
2 CU-8	Cube	Oh
3 SAPR-8	Square antiprism	D4d
4 TDD-8	Triangular dodecahedron	D2d
5 JGBF-8	Johnson – Gyrobifastigium (J26)	D2d

6 JBTP-8	Johnson – Biaugmented trigonal prism (J50)	C2v
7 BTPR-8	Biaugmented trigonal prism	C2v
8 JSD-8	Snub disphenoid (J84)	D2d
9 TT-8	Triakis tetrahedron	Td

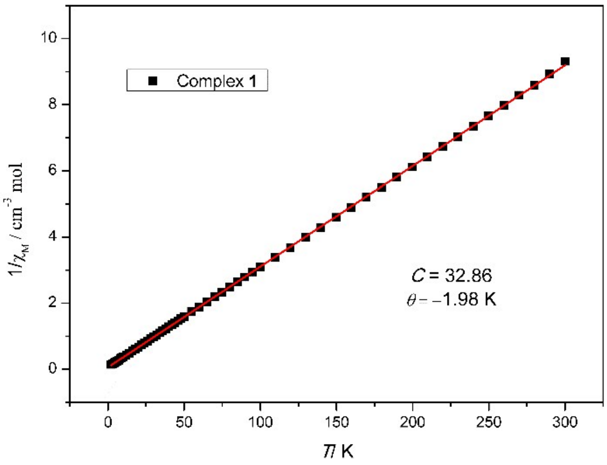

Fig. S6 Plot of $1/\chi_M$ versus *T* for **1**, the linear fit is the Curie-Weiss law fit at 1 kOe field.

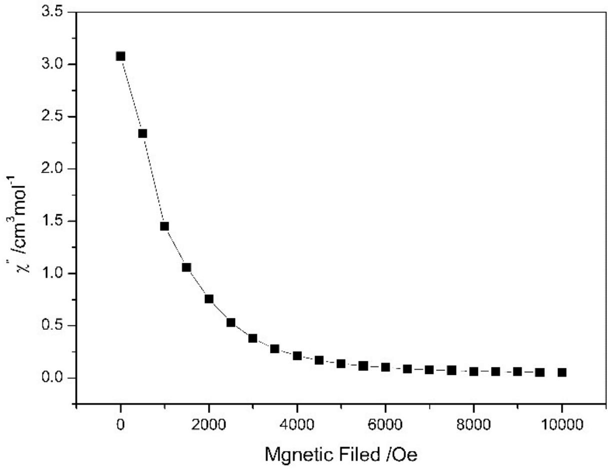

Fig. S7 Temperature dependence of the out-of-phase (χ′′) ac susceptibility for **3** under zero dc field at 1000 Hz.

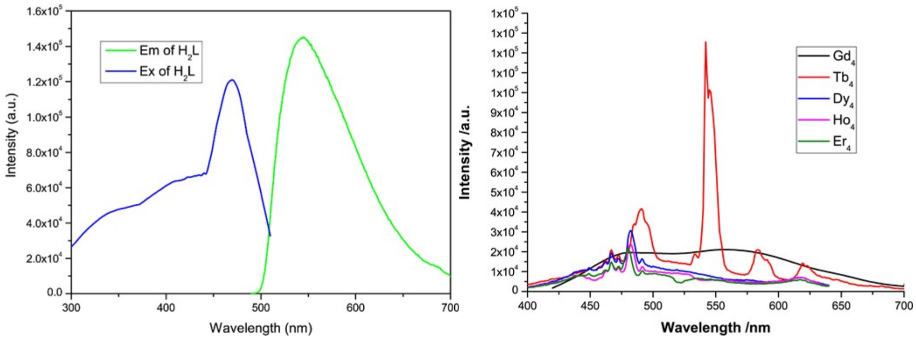

(a) (b)

Fig. S8 (a) The excitation and emission spectra of H_2L ligand. (b) Emission spectra of **1-5** in solid state at room temperature.

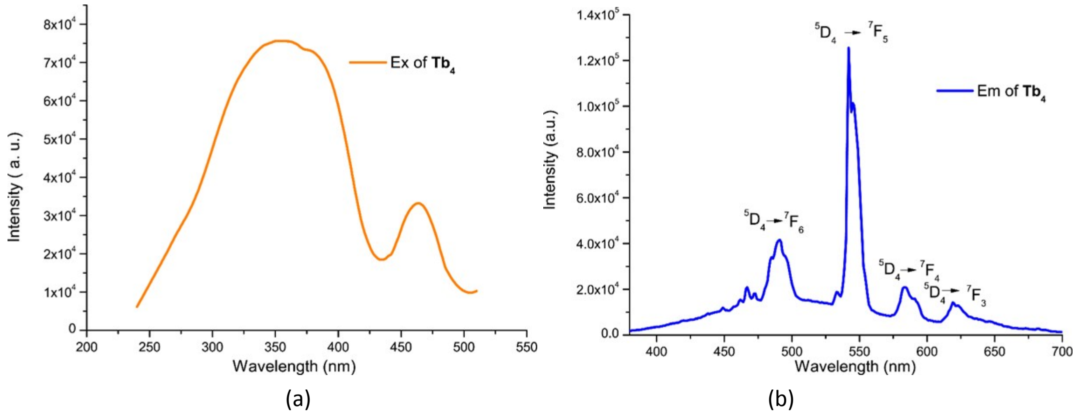

Fig. S9 Excitation spectrum (a) and emission spectrum (b) of **2** in solid state at room temperature.

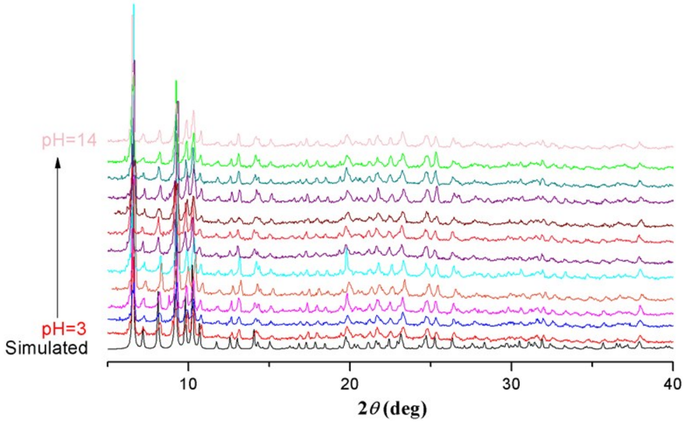

Fig. S10 PXRD patterns for the simulated and experimental samples of **2** soaked in aqueous solutions with pH values in the range of 3-14 for two days.

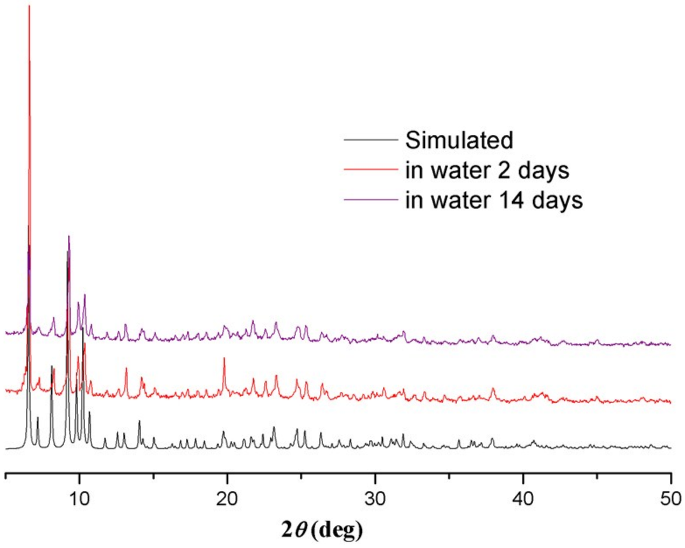

Fig. S11 PXRD patterns for the simulated and experimental samples of **2** soaked in water for 2 days and 14 days.

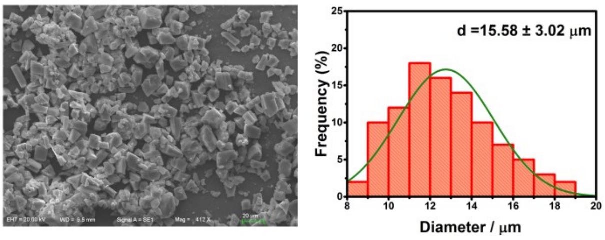

(a)

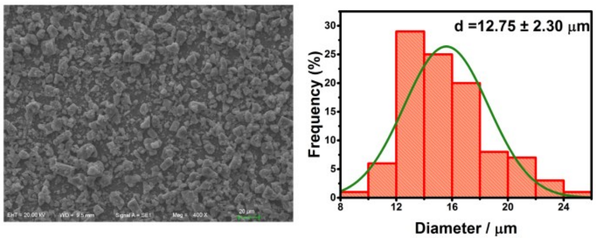

(b)

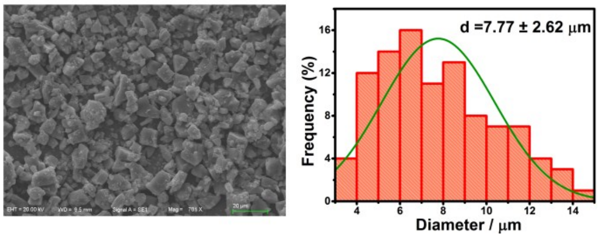

(c)

Fig. S12 SEM images (left) and particle size distributions of **2** (right) after being sonicated for 8 (a), 15 (b) and 30 (c) minutes.

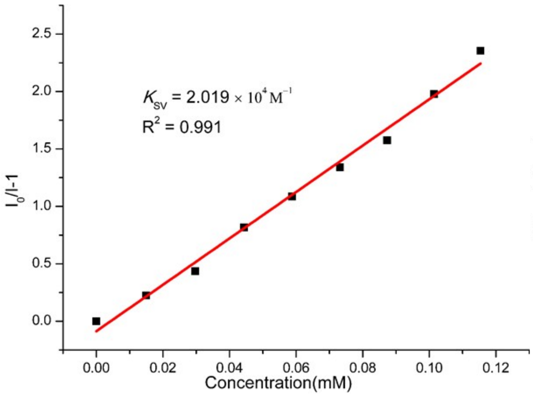

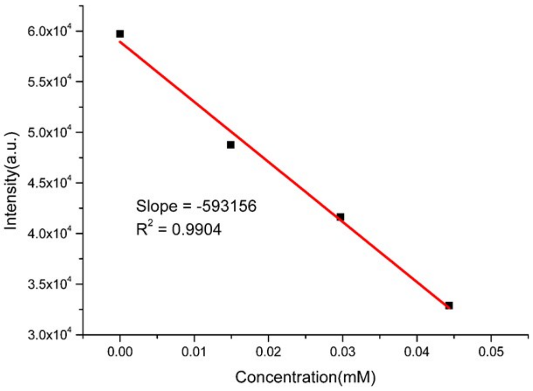

(a) (b)

Fig. S13 (a) Stern-Volmer plot of I_0/I-1 versus low Fe^{3+} concentration in the aqueous suspension of **2**. (b) Linear region of fluorescence intensity for the suspensions of **2** in water upon incremental addition of Fe^{3+} solutions.

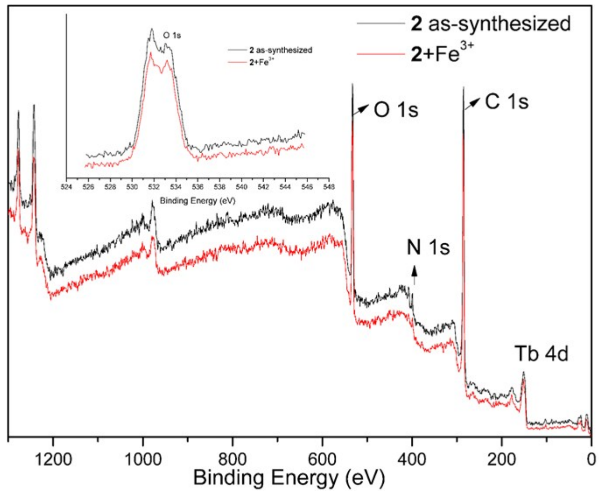

Fig. S14 Comparison of XPS spectra of **2** before (black) and after (red) its immersion in the Fe^{3+} aqueous solution.

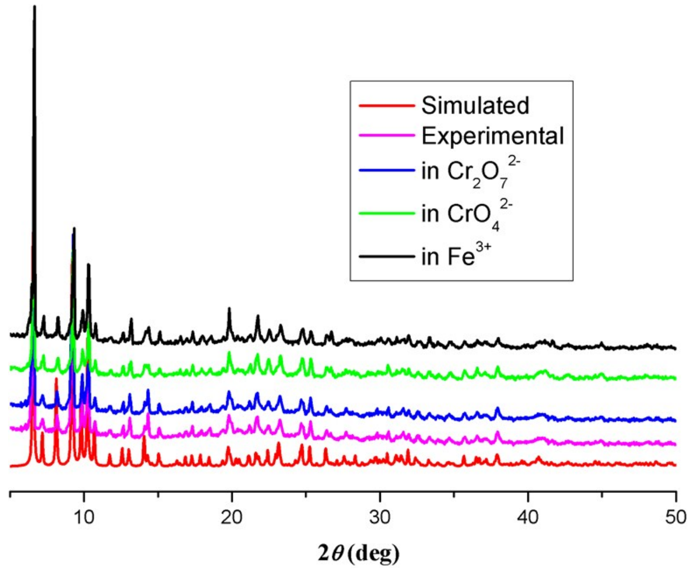

Fig. S15 PXRD patterns for **2** after the detection of Fe^{3+}, CrO_4^{2-} and $Cr_2O_7^{2-}$ ions.

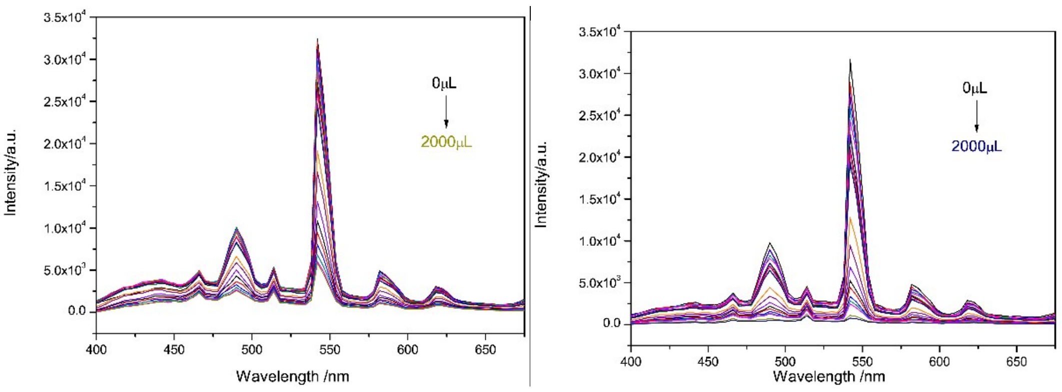

(a) (b)

Fig. S16 Luminescent responses of a water suspension of **2** (2 mg/2 mL) towards different concentrations of (a) CrO_4^{2-} ions (2×10^{-3} M, 0 μL-2000 μL) and (b) $Cr_2O_7^{2-}$ (2×10^{-3} M, 0 μL-2000 μL).

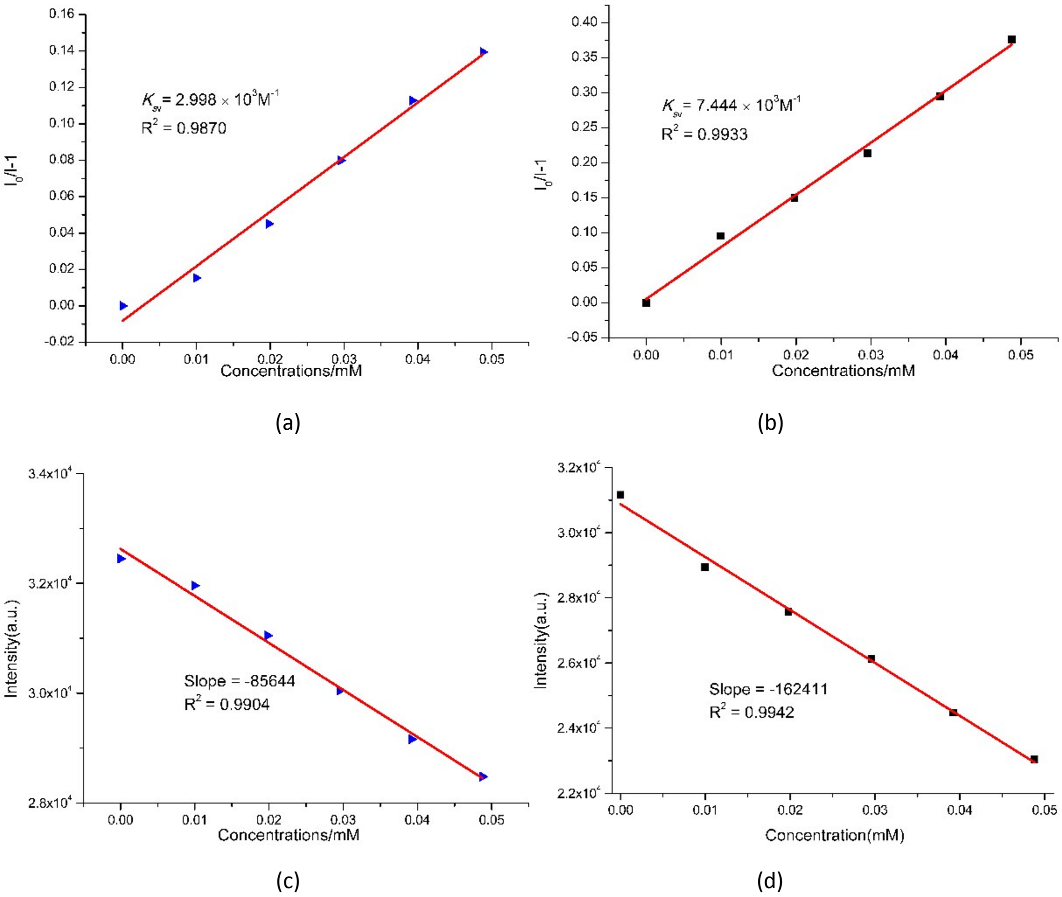

Fig. S17 Stern-Volmer plot of I_0/I-1 versus low concentration of CrO_4^{2-} (a) and $Cr_2O_7^{2-}$ (b) in the aqueous suspension of **2**, and linear region of fluorescence intensity for the suspensions of **2** in water upon incremental addition of CrO_4^{2-} (c) or $Cr_2O_7^{2-}$ (d) solutions.

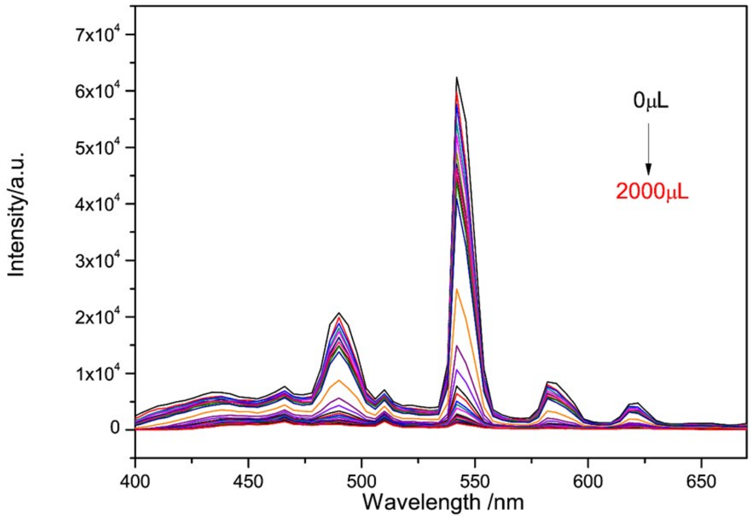

Fig. S18 Luminescence responses of an ethanol suspension of **2** (2 mg/2 mL) towards different concentrations of 4-NA (1 × 10^{-3} M, 0 μL-2000 μL).

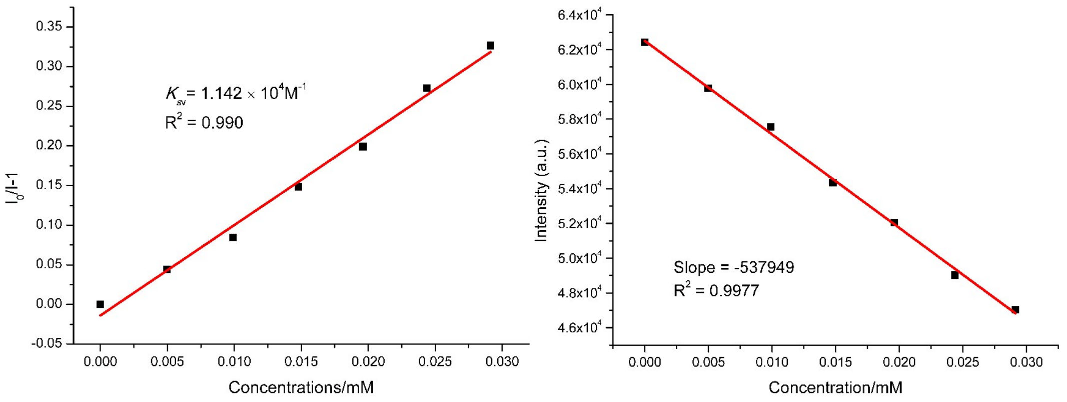

Fig. S19 (a) Stern-Volmer plot of I_0/I-1 versus low concentration of 4-NA in the ethanol suspension of **2**. (b) Linear region of fluorescence intensity for the suspensions of **2** in ethanol upon incremental addition of 4-NA solutions.

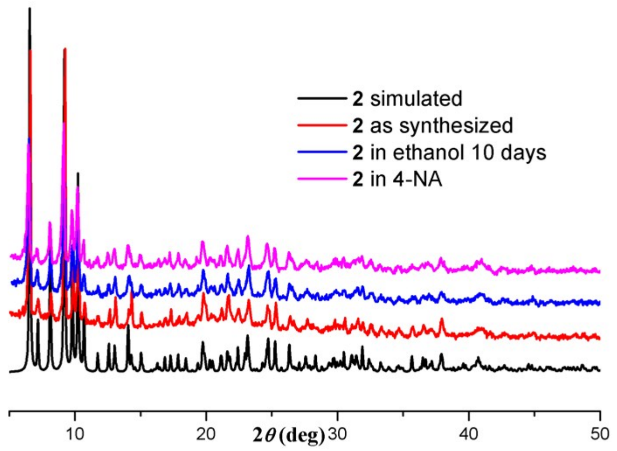

Fig. S20 PXRD patterns for **2** after the detection of 4-NA and being soaked in ethanol for 10 days

Table S3 The comparison of various Ln-complexes fluorescent sensors for Fe^{3+}, CrO_4^{2-} and $Cr_2O_7^{2-}$

Ln based complexes	Analyte	Quenching constant (K_{SV}, M^{-1})	Detection limits	Solvent	Ref
$[Tb_4L_4(NO_3)_2(Piv)_4]\cdot 2CH_3OH$	Fe^{3+}	1.86 × 10^4	10μM	water	This work
	CrO_4^{2-}	2.998 × 10^3	52μM		
	$Cr_2O_7^{2-}$	7.44 × 10^3	27μM		
$\{[Tb_2(Ccbp)_3\cdot 6H_2O]\cdot 3Cl^-\cdot 4H_2O\}$	Fe^{3+}	1.143 × 10^5		ethanol	1
$\{[Eu(L1)(BPDC)_{1/2}(NO_3)]\cdot H_3O\}_n$	Fe^{3+}	5.16 × 10^4		DMF	2
$\{[Tb(L1)(BPDC)_{1/2}(NO_3)]\cdot H_3O\}_n$	Fe^{3+}	4.30 × 10^4		DMF	2
$\{[Eu(1,5\text{-}Nds)_{0.5}(ox)(phen)(H_2O)]\cdot H_2O\}_n$	Fe^{3+}			water	3
$\{[Eu_2(MFDA)_2(HCOO)_2(H_2O)_6]\cdot H_2O\}_n$	Fe^{3+}		0.33 μM	DMF	4
$[(CH_3)_2NH_2]\cdot[Tb(bptc)]\cdot$xsolvents	Fe^{3+}		18.01 μM	ethanol	5
$[Tb(TBOT)(H_2O)](H_2O)_4(DMF)(NMP)_{0.5}$	Fe^{3+}	5.51 × 10^3	130 μM	water	6
	$Cr_2O_7^{2-}$	1.37 × 10^4	340 μM		
$[Eu(L2)(HCOO)(H_2O)]_n$	CrO_4^{2-}	1.5374× 10^3	1.2 μM	Water	7
	$Cr_2O_7^{2-}$	2.7626× 10^3	1.0 μM		
$[Tb(L2)(HCOO)(H_2O)]_n$	CrO_4^{2-}	1.3070× 10^3	1.8 μM	Water	7
	$Cr_2O_7^{2-}$	2.1335× 10^3	2.1 μM		

$[Eu_2(tpbpc)_4\cdot CO_3\cdot 4H_2O]\cdot$DMF·solvent	CrO_4^{2-}	4.85×10³	0.33 ppm	water	8
	$Cr_2O_7^{2-}$	1.04×10⁴	1.07 ppm		
$\{[Eu_2(L3)_{1.5}(H_2O)_2EtOH]\cdot DMF\}_n$	Fe^{3+}	2.942×10³	10 μM	DMF	9
	$Cr_2O_7^{2-}$	1.526×10³	10 μM		
$\{[Tb(TATAB)(H_2O)_2]\cdot NMP\cdot H_2O\}_n$	Fe^{3+}	3667	1 μM	water	10
	$Cr_2O_7^{2-}$	11106	5 μM		
$[Eu(Hpzbc)_2(NO_3)]\cdot H_2O$	$Cr_2O_7^{2-}$		22 μM	ethanol	11
$[Eu(HL4)(H_2O)_2(NO_3)]\cdot NO_3$	Fe^{3+}	4.03×10⁴	43μM	water	12
	$Cr_2O_7^{2-}$	7.52×10⁴	17μM		
$[Tb(HL4)(H_2O)_2(NO_3)]\cdot NO_3$	Fe^{3+}	4.54×10⁴	16μM	water	12
	$Cr_2O_7^{2-}$	4.59 × 10⁴	25μM		
$[Eu(HPIDC)(m\text{-}bdc)\cdot 1.5H_2O]_n$	$Cr_2O_7^{2-}$	4.1 × 10⁴		water	13
$[Tb(HPIDC)(m\text{-}bdc)\cdot 1.5H_2O]_n$	$Cr_2O_7^{2-}$	6.1 × 10³		water	13

$Ccbp^-$= 4-carboxy-1-(4-carboxybenzyl)pyridinium; H_2L1 = 2,5-di(pyridin-4-yl)terephthalic acid, BPDC = biphenyl-4,4′-dicarboxylic acid; 1,5-Nds = 1,5-naphthalenedisulfonate disulfonate; ox = oxalate; phen = 1,10-phenanthroline; m-H_2bdc = 1,3-benzenedicarboxylic acid; H_4bzptc = benzophenone-3,3′,4′,4′-tetracarboxylic acid; H_3PIDC =2-(4-pyridyl)-1H-imidazole-4,5-dicarboxylic acid; H_2MFDA = 9,9-dimethyl-fluorene-2,7-dicarboxylic acid; H_4bptc = tetracarboxylic acid; NMP = N-methyl-2-pyrrolidone; H_3TBOT (2,4,6-tris[1-(3-carboxylphenoxy)ylmethyl]mesitylene); H_2L = 5-((2′-cyano-[1,1′-biphenyl]-4-yl)methoxy)isophthalic acid; H_2L3 = 5,5′-(carbonylbis(azanediyl))diisophthalic acid; Htpbpc = 4′-[4,2′;6′,4′′]-terpyridin-4′-yl-biphenyl-4-carboxylic acid; H_3TATAB = 4,4′,4′-*s*-triazine-1,3,5- triyltri-*p*-aminobenzoate acid; H_2pzbc = 3-(1H-pyrazol-3-yl) benzoic acid; H3DMPhIDC =2-(3,4-dimethylphenyl)-1H- imidazole-4,5-dicarboxylic acid; H_3TATAB =4,4′,4′-s-triazine-1,3,5-triyltri-m-aminobenzoic acid, H_3PIDC = 2-(4-pyridyl)-1H- imidazole-4,5-dicarboxylic acid; m-H_2bdc = 1,3-benzenedicarboxylic acid; DMF = N,N-dimethylformamide

Table S4 The comparison of various coordination complexes fluorescent sensors for 4-NA

Coordination complex	Quenching constant (K_{SV}, M^{-1})	Detection limits	λex(nm)	λem(nm)	Solvent	Ref
$[Tb_4L_4(NO_3)_2(Piv)_4]\cdot 2CH_3OH$	1.14 × 10⁴	8.5μM	360	544	ethanol	This work
$[Cd_{1/2}(L5)_{1/3}(bib)_{1/2}(H_2O)]_n$	6.6 × 10⁴			325	DMSO	14
$\{[Cd_{1/2}(HL5)_2(bibp)_2]\cdot 3H_2O\}_n$	1.1 × 10⁴			375	DMSO	14
$[Cd_2(H_2L6)_2(H_2O)_5]\cdot 5H_2O\cdot 2DMF$	1.81 × 10⁴		340	480	isopropanol	15
$\{[Zn4(\mu 3\text{-}OH)_2(L7)(H_2O)_2]\cdot 2DMF\}_n$			350	400 red-shifted about 40 nm.	DMF	16
$[Zn(L8)_{0.5}(1,10\text{-}phen)(H_2O)]\cdot 2H_2O$	6556		330	456	DMA	17
$[Zn(L8)_{0.5}(1,10\text{-}phen)(H_2O)]\cdot 2H_2O$	3955		318	396	DMA	17

H_3L5 = tris(p-carboxyphenyl)phosphane oxide; bib = 1,4-bis(imidazol-1-yl)benzene bibp = 4,4′-bis(imidazol- 1-yl)biphenyl; H_4L6 = 2,5-bis-(3,5-dicarboxyphenyl)thiopheneamide; H_6L7 = [1,1′;4′,1′′]terphenyl-3,5,2′,5′,3′′,5′′-hexacarboxylic acid; H_4L8 =bis-(3,5-dicarboxyphenyl)terephthalamide; DMA = N,N-dimethylacetamide; DMSO = dimethyl sulfoxide

References

S1. K.-M. Wang, L. Du, Y.-L. Ma, J.-S. Zhao, Q. Wang, T. Yan and Q.-H. Zhao, *CrystEngComm*, 2016, **18**, 2690-2700.

S2. W. Yan, C. Zhang, S. Chen, L. Han and H. Zheng, *ACS Appl. Mater. Interfaces*, 2017, **9**, 1629-1634.

S3. R. Li, X.-L. Qu, Y.-H. Zhang, H.-L. Han and X. Li, *CrystEngComm*, 2016, **18**, 5890-5900.

S4. X. H. Zhou, L. Li, H. H. Li, A. Li and W. Huang, *Dalton Trans.*, 2013, **42**, 12403-12409.

S5. X. L. Zhao, D. Tian, Q. Gao, H. W. Sun, J. Xu and X. H. Bu, *Dalton Trans*., 2016, **45**, 1040-1046.

S6. M. Chen, W. -M. Xu, J. -Y. Tian, H. Cui, J.-X. Zhang, C. -S. Liu and M. Du, *J. Mater. Chem. C*, 2017, **5**, 2015-2021.

S7. Z. Sun, M. Yang, Y. Ma and L. Li, *Cryst. Growth Des.*, 2017, **17**, 4326-4335.

S8. J. Liu, G. Ji, J. Xiao and Z. Liu, *Inorg. Chem.*, 2017, **56**, 4197-4205.

S9. W. Liu, X. Huang, C. Xu, C. Chen, L. Yang, W. Dou, W. Chen, H. Yang and W. Liu, *Chem. –Eur. J.*, 2016, **22**, 18769-18776.

S10. G. X. Wen, M. L. Han, X. Q. Wu, Y. P. Wu, W. W. Dong, J. Zhao, D. S. Li and L. F. Ma, *Dalton Trans.*, 2016, **45**, 15492-15499.

S11. G. P. Li, G. Liu, Y. Z. Li, L. Hou, Y. Y. Wang and Z. Zhu, *Inorg. Chem.*, 2016, **55**, 3952-3959.

S12. W. Gao, F. Liu, B. Y. Zhang, X. M. Zhang, J. P. Liu, E. Q. Gao and Q. Y. Gao, *Dalton Trans.*, 2017, **46**, 13878-13887.

S13. X. H. Huang, L. Shi, S. M. Ying, G. Y. Yan, L. H. Liu, Y. Q. Sun and Y. P. Chen, *CrystEngComm*, 2018, **20**, 189-197.

S14. L. Huo, J. Zhang, L. Gao, X. Wang, L. Fan, K. Fang and T. Hu, *CrystEngComm*, 2017, **19**, 5285-5292.

S15. F. Wang, Z. Yu, C. Wang, K. Xu, J. Yu, J. Zhang, Y. Fu, X. Li and Y. Zhao, *Sen. Actuators B Chem.*, 2017, **239**, 688-695.

S16. X.-Y. Wan, F.-L. Jiang, C.-P. Liu, K. Zhou, L. Chen, Y.-L. Gai, Y. Yang and M.-C. Hong, *J. Mater. Chem. A*, 2015, **3**, 22369-22376.

S17. F. Wang, C. Wang, Z. Yu, Q. He, X. Li, C. Shang and Y. Zhao, *RSC Adv.*, 2015, **5**, 70086-70093.
